# Selection and Validation of Reference Genes for RT-qPCR Analysis of Lipid Biosynthesis-Related Genes in *Perilla frutescens*

**DOI:** 10.3390/plants15172588

**Published:** 2026-08-25

**Authors:** Siya Yue, Yuanlin Zhang, Ruiqi Tong, Haoyang Geng, Yingtao Xia, Sunyu Shi, Yueping Zheng, Zhifu Zheng, Yi Gan

**Affiliations:** 1 Jixian Honors College, Zhejiang A&F University, Hangzhou 311300, China; 202324060101@stu.zafu.edu.cn; 2 College of Advanced Agricultural Sciences, Zhejiang A&F University, Hangzhou 311300, China; senlinpengyou@stu.zafu.edu.cn (Y.Z.); tongruiqi77@163.com (R.T.); ghy741858672@163.com (H.G.); 202320020231@zafu.edu.cn (Y.X.); zhengyp@zafu.edu.cn (Y.Z.);

**Keywords:** *Perilla frutescens*, reference gene selection, RT-qPCR, lipid biosynthesis, expression stability

## Abstract

*Perilla frutescens* is a specialty oilseed crop rich in polyunsaturated fatty acids, particularly α-linolenic acid (ALA), and the molecular regulation of seed lipid metabolism is an active area of research. Reverse-transcription quantitative PCR (RT-qPCR) is essential for investigating the molecular mechanisms underlying seed oil biosynthesis; however, systematically validated reference genes are lacking for this species. Based on *P. frutescens* transcriptome data, seven commonly used housekeeping genes—*PfActin*, *Pf18S* (18S pre-ribosomal assembly protein GAR2-like), *PfEF-1α*, *PfCYP*, *PfTUA*, *PfTUB*, and *PfGAPDH*—were initially selected. After *PfGAPDH* was excluded because of its extremely low abundance and unstable expression, the remaining six candidates were evaluated in vegetative organs (leaves, stems, and roots), seeds at different developmental stages, and leaves subjected to PEG 6000 treatment. Expression stability was independently assessed using geNorm, NormFinder, and BestKeeper, and the resulting stability values were integrated with RefFinder. *Pf18S* showed the highest overall stability in the complete sample set and developing seeds. Under osmotic stress, *PfTUB* was the most stable single reference gene, and geNorm identified *PfTUB* + *Pf18S* as the optimal two-gene combination. In vegetative organs, *PfTUB* ranked first and *PfTUA* second, whereas *PfEF-1α* was among the least stable genes in most sample sets. The recommended two-gene combinations were *PfTUA* + *PfTUB* for vegetative organs, *Pf18S* + *PfTUA* for developing seeds, and *PfTUB* + *Pf18S* for osmotic stress. Biological validation showed that normalization to *Pf18S* reproduced spatiotemporal expression patterns of six key lipid-biosynthesis genes that were consistent with the dynamic accumulation of total seed oil and ALA. These results support a tiered normalization strategy using *Pf18S* for preliminary screening and condition-specific reference-gene pairs for precise quantification.

## 1. Introduction

Plant oils are increasingly important renewable resources for bioenergy, industrial feedstocks, and human nutrition, and the development and utilization of specialty oilseed crops are therefore of considerable interest. Among oilseed plants, *Perilla frutescens* has attracted particular attention because its seeds are rich in polyunsaturated fatty acids (PUFAs) [1]. Perilla is a traditional economic crop with food, medicinal, and industrial uses. Its seeds generally contain more than 40% oil, and the oil content of selected high-yielding cultivars can reach 46–58% [1,2]. α-Linolenic acid (ALA) is especially abundant in perilla seed oil, typically accounting for more than 60% of total fatty acids, making perilla one of the richest natural sources of ALA [2,3]. A substantial body of evidence indicates that ALA and its metabolites have anti-inflammatory and lipid-lowering effects and may contribute to the prevention of cardiovascular disease, thereby enhancing the value of perilla seed oil for functional foods and pharmaceutical applications [3,4].

Recent advances in perilla genomics and transcriptomics have enabled the identification and functional characterization of numerous enzymes and transcription factors involved in fatty-acid biosynthesis. Consequently, elucidating the regulatory network underlying the synthesis and accumulation of high levels of PUFAs in perilla seeds has become a major research focus [5,6]. In the desaturation pathway, several genes encoding ω-3 fatty-acid desaturases, including *PfFAD3*, *PfFAD7*, and *PfFAD8*, have been cloned and functionally validated in heterologous systems, and their expression is induced by environmental factors such as low temperature [7,8,9]. In triacylglycerol (TAG) assembly, the glycerol-3-phosphate acyltransferase gene *PfGPAT9* and members of the diacylglycerol acyltransferase family (*PfDGATs*) are positively associated with rapid oil accumulation [10,11]. Transcription-factor families, including AP2/EREBP (e.g., *PfWRI1-10*), Dof (e.g., *PfDof29*), bZIP (e.g., *PfbZIP85*), and MADS-box proteins, also participate extensively in the transcriptional regulation of perilla lipid biosynthesis [12,13,14,15,16]. Together, these studies have substantially advanced our understanding of the molecular basis of seed oil synthesis and accumulation in perilla.

RT-qPCR is a key method for investigating complex metabolic networks and gene function in plants, but its accuracy depends on rigorous experimental quality control and appropriate normalization. The MIQE (Minimum Information for Publication of Quantitative Real-Time PCR Experiments) guidelines and their subsequent updates emphasize that RT-qPCR studies should systematically report sample processing, primer specificity, amplification efficiency, and data-normalization procedures to ensure reproducibility and reliability [17]. The selection of stably expressed reference genes is a fundamental prerequisite for normalization. However, numerous plant studies have demonstrated that conventional housekeeping genes, such as *Actin* and *GAPDH*, are not constitutively expressed under all experimental conditions. Their stability can vary markedly among species, tissues, developmental stages, and stress treatments. Normalization using a single unvalidated gene can therefore introduce substantial quantitative bias and may even produce conclusions that contradict the underlying biology [17,18,19]. Reference genes should not be selected solely from the literature or transferred between species on the basis of homology; instead, their stability must be empirically evaluated and validated for each specific experimental system, including the plant species, tissue type, developmental stage, and environmental condition. This principle is now widely recognized as essential for ensuring the quality of plant molecular-biology data and the reliability of the resulting conclusions. Given that *Perilla frutescens* is an allotetraploid with multiple homologous gene copies—such as *PfFAD2* and *PfFAD3*—the stability of reference genes may be further influenced by transcriptional divergence among these copies, thereby adding an additional layer of complexity to the selection of appropriate normalization controls.

RT-qPCR has been used extensively in previous perilla studies to analyze the expression of lipid-metabolism genes [5,7,10,11], yet systematic validation of reference genes across tissues, developmental stages, and osmotic stress remains limited. In those studies, conventional housekeeping genes such as *Actin* or 18S rRNA were commonly adopted as normalization controls without prior stability testing under diverse experimental conditions. This practice is problematic because perilla is an allotetraploid with multiple homologous gene copies, which can affect reference-gene stability. More importantly, reference-gene stability is known to be species- and condition-specific [17,18,19], meaning that recommendations from model plants or related taxa cannot be directly transferred to perilla. To address this, we employed a multi-algorithm approach combining geNorm, NormFinder, BestKeeper, and RefFinder to systematically evaluate six candidate reference genes across vegetative organs, developing seeds, and osmotic-stress conditions, thereby establishing a robust, condition-specific normalization framework. Specifically, seven housekeeping genes (*PfActin*, *Pf18S*, *PfEF-1α*, *PfCYP*, *PfTUA*, *PfTUB*, and *PfGAPDH*) were selected from transcriptome data and tested in leaves, stems, roots, five seed developmental stages, and PEG-treated leaves. The three algorithms were applied independently and integrated via RefFinder. The selected reference genes were further validated using six genes involved in TAG assembly, fatty-acid desaturation, and acyl editing (*PfDGAT*, *PfPDAT*, *PfFAD2*, *PfFAD3*, *PfLPCAT1.1*, and *PfLPCAT1.2*). This work provides a methodological foundation for accurate gene-expression quantification and further investigation of PUFA metabolism in perilla seeds.

## 2. Results

### 2.1. PCR Specificity and Amplification Efficiency of the Candidate Reference Genes

**Figure 1:**
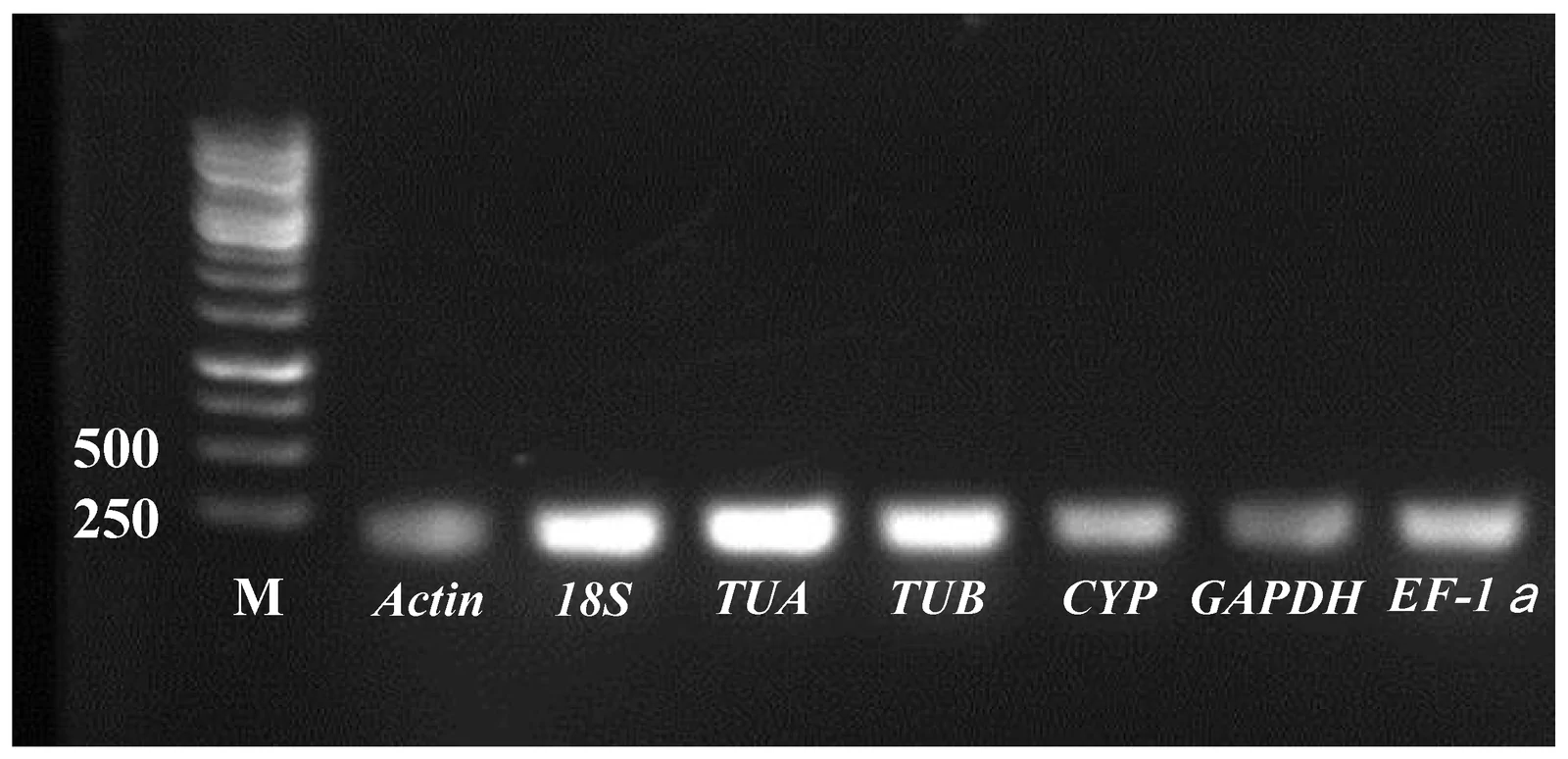
Agarose-gel electrophoresis showing the specificity of PCR amplification for seven candidate reference genes in *Perilla frutescens*.

**Figure 2:**
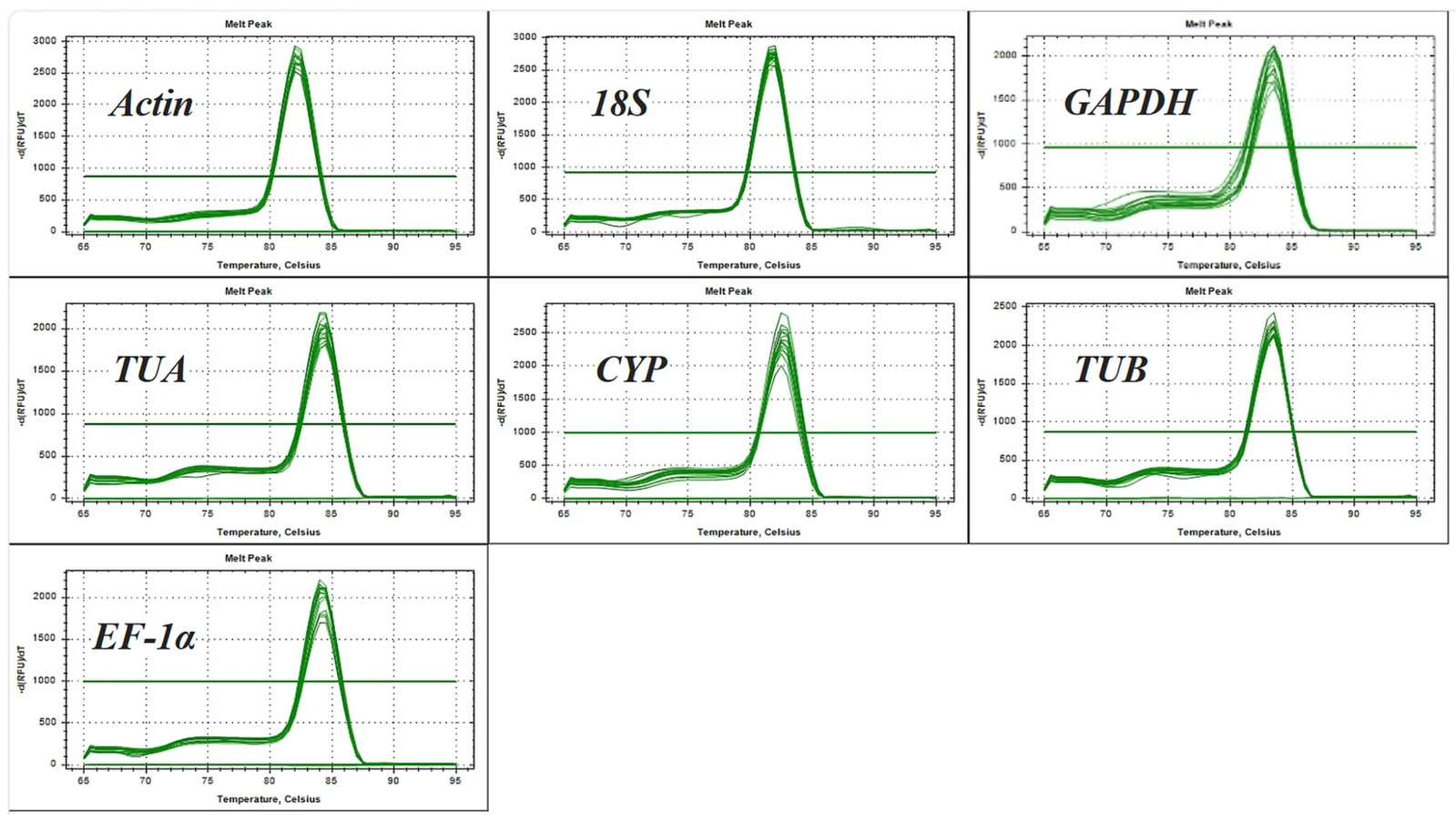
Melting-curve analysis of seven candidate reference genes in *Perilla frutescens*.

**Figure 3:**
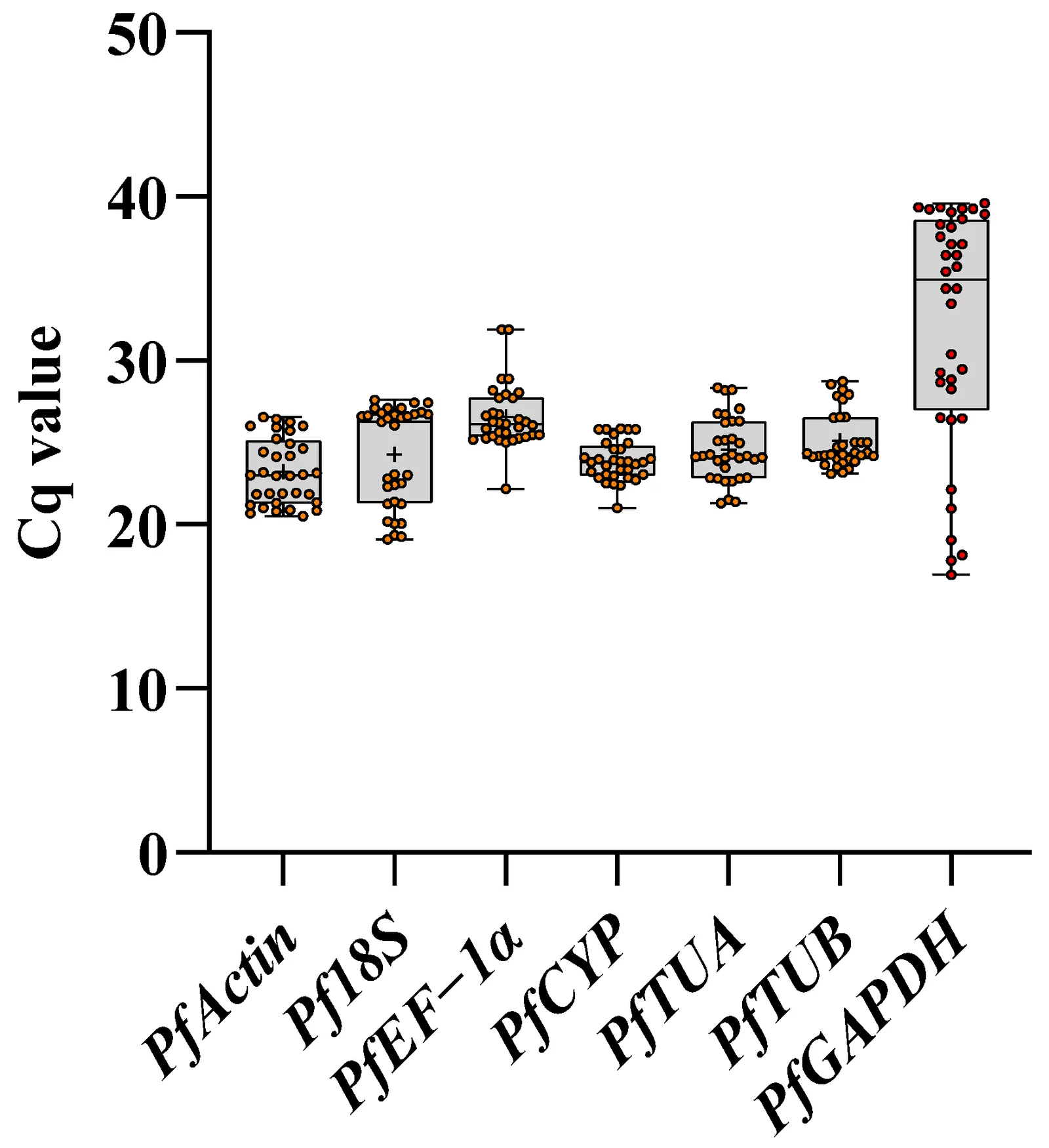
Distribution of Cq values for candidate reference genes in *Perilla frutescens*. Vertical boxplots (25th–75th percentiles, median, min–max) are overlaid with individual data points. Red dots highlight PEG 6000 treatment, showing that *PfGAPDH* expression instability was primarily induced by osmotic stress.

To identify reference genes suitable for RT-qPCR analysis in perilla, seven housekeeping genes (*PfActin*, *Pf18S* (18S pre-ribosomal assembly protein GAR2-like), *PfEF-1α*, *PfCYP*, *PfTUA*, *PfTUB*, and *PfGAPDH*) were selected from transcriptome data (Table 1). PCR amplification using pooled cDNA produced a single clear band of the expected size for each candidate gene (Figure 1), indicating high primer specificity. Primer specificity and amplification performance were further evaluated by RT-qPCR melting-curve analysis and serial-dilution standard curves. Each candidate reference gene produced a single sharp melting peak (Figure 2), confirming specific amplification. As shown in Table 1, the coefficients of determination (R^2^) for the seven primer pairs ranged from 0.9900 to 0.9995, indicating excellent linearity across the five-step dilution series. Amplification efficiencies ranged from 91.22% to 101.47%, within the 90–110% range recommended by the MIQE guidelines, and were therefore suitable for subsequent stability analysis. The standard-curve equations, slopes, and raw Cq values for the dilution series are provided in Supplementary Material Table S3. The distribution of Cq values across all samples revealed a clear limitation of *PfGAPDH* (Figure 3; the complete raw Cq matrix is provided in Supplementary Material Table S4). Whereas the other six candidate genes showed Cq values predominantly between 20 and 28, *PfGAPDH* had a median Cq of approximately 36 and varied markedly among tissues and PEG treatments. These results indicate that *PfGAPDH* was expressed at very low and highly variable levels, particularly under osmotic stress. Because this behavior is inconsistent with the requirements for a reference gene, *PfGAPDH* was excluded, and the remaining six genes were retained for further analysis.

### 2.2. Multi-Algorithm Evaluation of Candidate Reference-Gene Stability

The expression stability of the six retained candidate reference genes was evaluated in different vegetative organs, developing seeds, and PEG 6000-treated leaves using geNorm, NormFinder, and BestKeeper. The final integrated stability scores were obtained with RefFinder (Supplementary Material Table S4).

**Figure 4:**
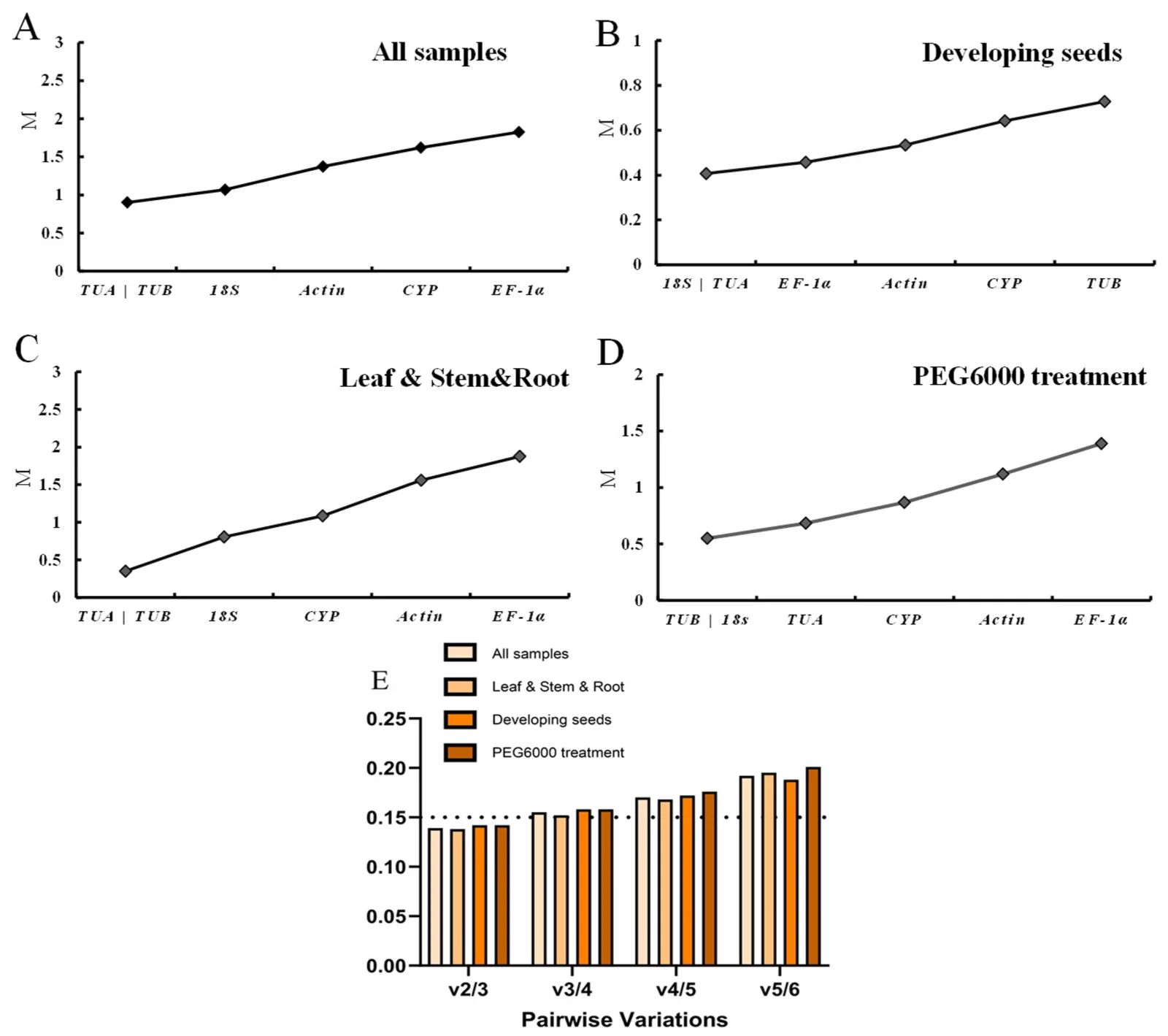
geNorm analysis of expression-stability values (M; (**A**–**D**)) and pairwise variation (V_n_/V_n+1_; (**E**)) for six candidate reference genes. (**A**) All samples; (**B**) developing seeds; (**C**) vegetative organs (leaves, stems, and roots); (**D**) PEG 6000-induced osmotic stress; and (**E**) pairwise-variation analysis used to determine the optimal number of reference genes. The dashed line in (**E**) indicates the recommended threshold of 0.15. In panels (**A**–**D**), the x-axis represents genes arranged in ascending order of their M values (from most stable to least stable) within each independent sample set. The symbol “|” indicates that the two adjacent genes share the same M value and thus are ranked equally. The lines connecting the data points are intended only to assist visual comparison of the stability gradient; they do not imply continuity of the x-axis variable, which is categorical.

In the geNorm analysis, lower average expression-stability (M) values indicate greater stability. The six candidate genes exhibited clear condition-dependent differences across the four sample sets: all samples, vegetative organs (leaves, stems, and roots), developing seeds, and PEG 6000-treated leaves (Figure 4A–D). Except for *PfCYP* and PfEF-1α in the complete sample set and *PfActin* and *PfEF-1α* in vegetative organs, the M values of the candidate genes were below the commonly used threshold of 1.5, indicating generally acceptable stability under most conditions. *PfTUA* and *PfTUB* were the most stable genes in the complete sample set and vegetative organs (Figure 4A,C). *Pf18S* and *PfTUA* formed the most stable pair in developing seeds (Figure 4B), whereas *PfTUB* and *Pf18S* were the most stable pair under osmotic stress (Figure 4D). By contrast, *PfTUB* had the highest M value in developing seeds, and *PfEF-1α* was the least stable gene in the other three sample sets (Figure 4A,C,D). The optimal number of reference genes was further assessed using geNorm pairwise variation (V_n_/V_n+1_; Figure 4E). Under the conventional geNorm interpretation, V_n_/V_n+1_ < 0.15 indicates that inclusion of an additional reference gene provides only a limited reduction in normalization-factor variation. In all four sample sets, V2/3 was below 0.15, whereas V3/4, V4/5, and V5/6 exceeded this reference threshold. Thus, within the current candidate-gene pool, adding a third or additional reference gene would provide limited marginal improvement while increasing experimental cost and workload. Two-gene combinations of the most stable candidates were therefore selected as the preferred compromise between normalization accuracy and experimental efficiency.

NormFinder ranks genes according to a model-based stability value (SV), with lower values indicating greater stability (Table 2). *Pf18S* had the lowest SV and was the most stable gene when all samples were analyzed together. In vegetative organs, *PfTUB* and *PfTUA* had the two lowest SVs. In PEG 6000-treated leaves, *PfTUB* and *PfActin* were the most stable candidates. In developing seeds, NormFinder ranked *PfEF-1α* first and *Pf18S* second. The partial inconsistency between this result and the geNorm and integrated rankings suggests that the weighting of intergroup variance in a single model can influence stability estimates for grouped developmental samples, highlighting the value of combining multiple algorithms.

BestKeeper evaluates stability using the standard deviation (SD) and coefficient of variation (CV) of the raw Cq values (Table 3). In the complete sample set, *PfCYP* had the lowest SD, followed by *PfEF-1α* and *Pf18S*. In vegetative organs, *PfTUA* and *PfTUB* had the lowest SD values, followed by *Pf18S*. *Pf18S* ranked second in developing seeds. Under PEG 6000 treatment, *Pf18S* and *PfTUB* shared the lowest SD value (0.32), further supporting their stability under this condition.

**Figure 5:**
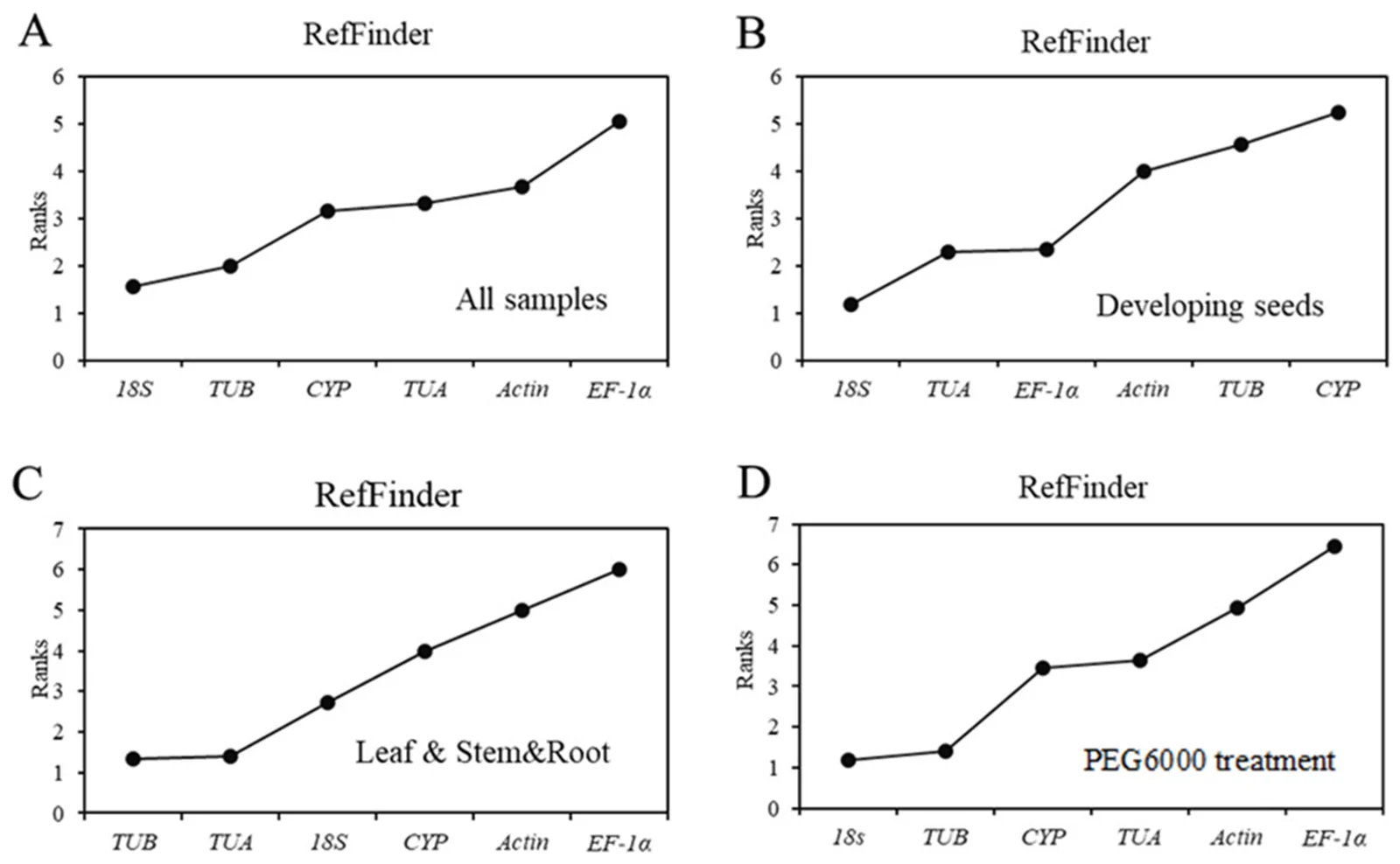
RefFinder integrated stability scores of six candidate reference genes across different sample sets. (**A**) All samples; (**B**) developing seeds; (**C**) vegetative organs (leaves, stems, and roots); (**D**) PEG 6000-induced osmotic stress. The x-axis represents genes arranged in order of their integrated stability scores (from most stable to least stable) within each independent sample set. The lines connecting the data points are intended only to assist visual comparison of the stability gradient; they do not imply continuity of the x-axis variable, which is categorical.

To integrate the outputs of the three independent algorithms and reduce model-specific bias, RefFinder was used to calculate the geometric mean of the stability scores (Figure 5). *Pf18S* ranked first in the complete sample set (Figure 5A), developing seeds (Figure 5B), and PEG 6000-treated leaves (Figure 5D), demonstrating the greatest overall stability. In vegetative organs, *PfTUB* ranked first (Figure 5C). *PfEF-1α* consistently ranked near the bottom in most sample sets and is therefore unsuitable as a normalization gene for perilla RT-qPCR analyses. When the RefFinder stability scores were considered together with geNorm pairwise variation, the following condition-specific two-gene combinations were recommended: *PfTUA* + *PfTUB* for vegetative organs, *Pf18S* + *PfTUA* for developing seeds, and *PfTUB* + *Pf18S* for osmotic-stress experiments. These algorithm-based analyses established the mathematical stability of the candidate genes. Because the ultimate purpose of reference-gene selection is accurate quantification in a defined biological process, the selected genes were subsequently validated in the context of perilla seed lipid synthesis and accumulation.

### 2.3. Expression Patterns of Lipid-Biosynthesis Genes Normalized Using a Stable Reference Gene

**Figure 6:**
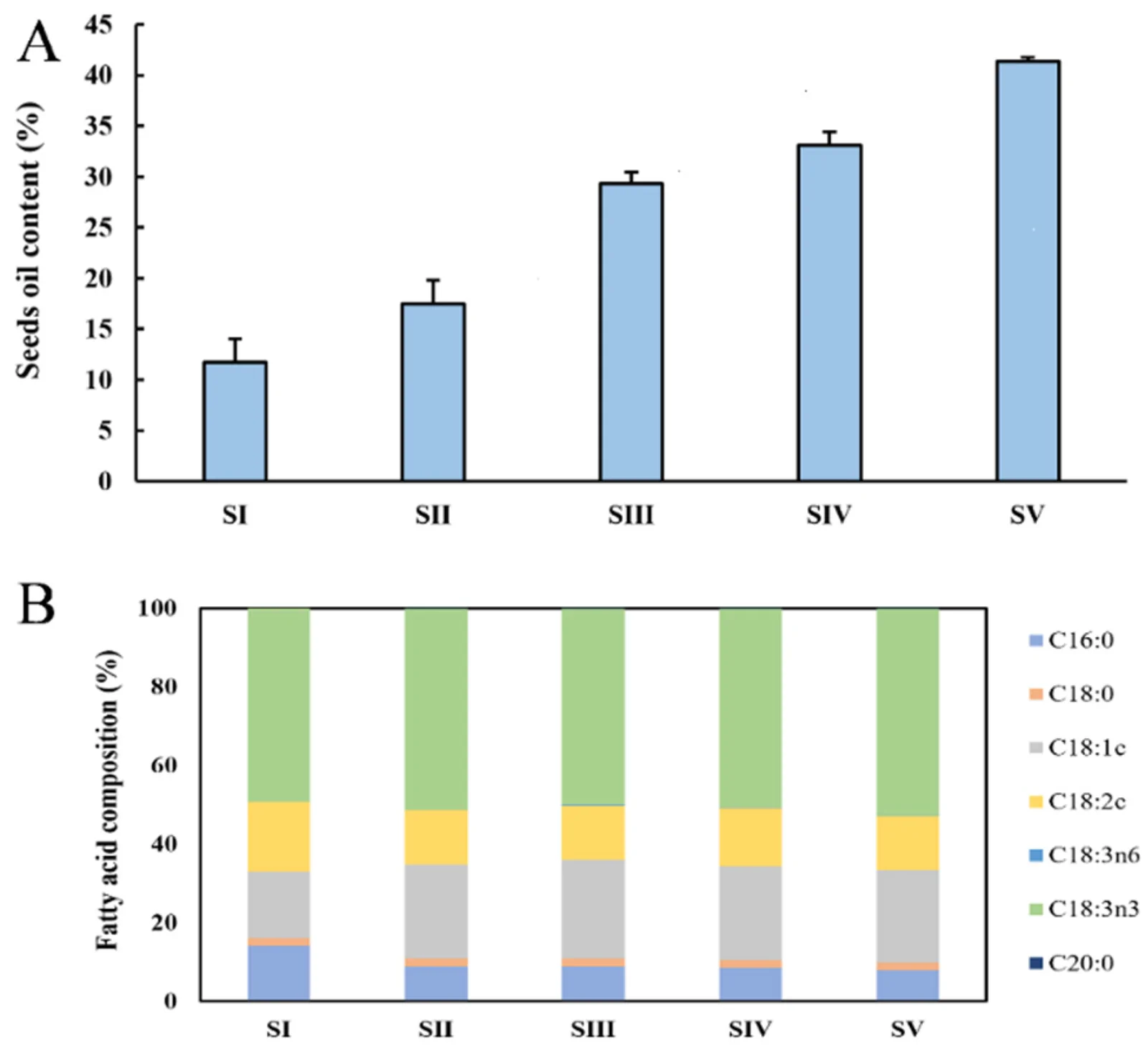
Accumulation dynamics of total oil content (**A**) and the relative proportions of major fatty acids (**B**) during *Perilla frutescens* seed development. SI–SV correspond to 6, 13, 19, 25, and 31 days after flowering, respectively.

The multi-algorithm evaluation provided a mathematical basis for selecting reference genes under different experimental conditions, but their practical value depends on whether they effectively reduce technical variation and accurately reflect target-gene expression dynamics. Rapid oil accumulation and ALA synthesis during seed development are central determinants of perilla seed quality. To evaluate the performance of the top-ranked gene *Pf18S* in lipid-metabolism studies, we conducted cross-validation using physiological accumulation profiles, transcriptome-based pathway information, and normalized target-gene expression. Total oil content and the relative abundance of major fatty acids were quantified by gas chromatography with flame-ionization detection (GC-FID) in seeds of the Gansu white perilla accession ZS6 at five developmental stages (SI–SV). Total seed oil content increased markedly during development and maturation (Figure 6A). Oil content was only 11.74% at SI, increased rapidly from SII to SIV, and reached 41.37% at maturity (SV). ALA (C18:3n3) was the predominant fatty acid at all stages, increasing from 49.28% of total fatty acids at SI to 52.67% at SV (Figure 6B). In contrast, palmitic acid (C16:0) decreased from 14.07% at SI to 7.95% at SV. The proportions of oleic acid (C18:1c) and linoleic acid (C18:2c) remained relatively stable during the middle and late stages (SII–SIV), at approximately 23–25% and 13–15%, respectively.

These physiological data provided a benchmark for evaluating the expression of key lipid-biosynthesis genes. Using the transcriptome dataset, we reconstructed the lipid-metabolism network spanning de novo fatty-acid synthesis in plastids, the Kennedy pathway and acyl editing in the endoplasmic reticulum, and TAG assembly. An accompanying heatmap summarized the expression of major genes and transcription factors, including *WRI1*, *FUS3*, *ABI3*, *KASI–III*, *SAD*, *FATA/B*, *GPAT*, *LPAAT*, *DGAT*, *PDAT*, *FAD2*, *FAD3*, and LPCAT (Supplementary Figure S2C). To assess normalization performance in RT-qPCR analyses, six representative target genes were selected: *PfDGAT* and *PfPDAT* for TAG assembly, *PfFAD2* and *PfFAD3* for fatty-acid desaturation, and *PfLPCAT1.1* and *PfLPCAT1.2* for acyl editing. Relative expression patterns were analyzed using *Pf18S* (*PfGAR2-like*), the highest-ranked single reference gene in the RefFinder analysis.

**Figure 7:**
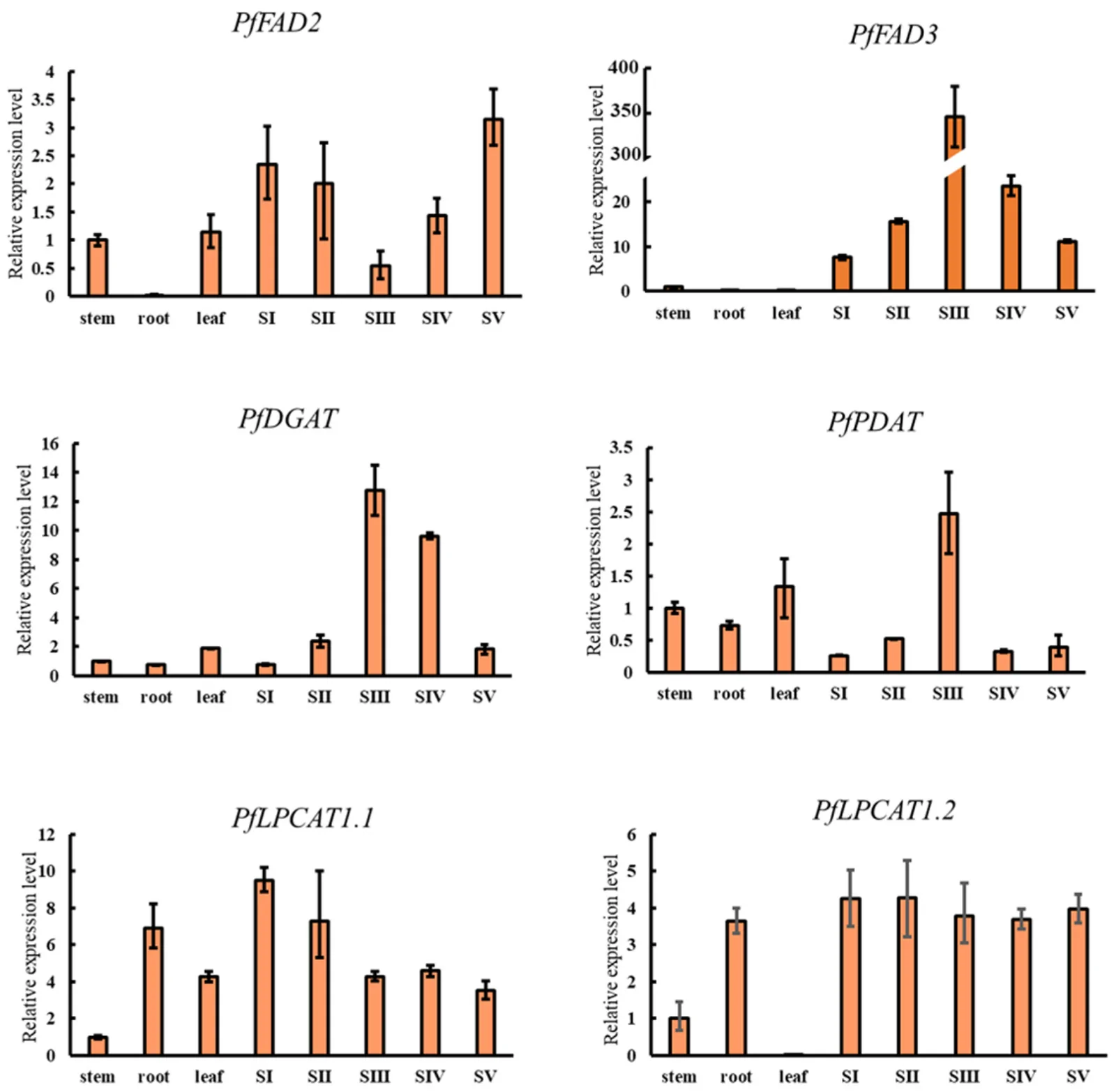
Relative expression patterns of six lipid-biosynthesis-related genes in different vegetative organs and seed developmental stages of *Perilla frutescens*, normalized using *Pf18S*. This figure illustrates qualitative expression trends during seed development using *Pf18S* as a representative single reference gene. The condition-specific two-gene combinations recommended by the multi-algorithm analysis (e.g., *Pf18S* + *PfTUA* for developing seeds) remain the preferred choice for studies requiring high quantitative precision.

*PfDGAT*, *PfPDAT*, and *PfFAD3* reached their highest expression levels at SIII (Figure 7). These expression peaks coincided with high-expression regions in the transcriptome heatmap (Supplementary Figure S2C) and with the steep increase in total oil content between SII and SIV (Figure 6A), suggesting that SIII may represent a period of particularly active TAG assembly and PUFA synthesis in perilla seeds. *PfFAD2* expression was highest at SV, suggesting a potential role in fine-tuning the proportion of unsaturated fatty acids during late oil accumulation. *PfLPCAT1.1* was most highly expressed at SI, which may be associated with active early-stage acyl editing and membrane-lipid metabolism, whereas *PfLPCAT1.2* remained relatively stable throughout development and may contribute to lipid homeostasis. The stage-specific expression patterns of these genes were broadly consistent with the dynamics of seed oil accumulation, supporting the utility of *Pf18S* for exploratory analysis of large-scale expression trends in lipid metabolism. This validation was intended to demonstrate the feasibility of using the most stable single reference gene for preliminary expression profiling; condition-specific two-gene combinations remain preferable for precise quantitative analyses.

## 3. Discussion

This study used a systematic screening strategy that combined three independent algorithms—geNorm, NormFinder, and BestKeeper—with the integrated RefFinder assessment. Seven housekeeping genes were initially evaluated, and *PfGAPDH* was excluded because of inadequate expression characteristics. The remaining six candidate genes showed stability values rankings among tissues, seed developmental stages, and osmotic-stress conditions, confirming that reference-gene performance is strongly condition dependent. *Pf18S* had the highest overall stability in the complete sample set, developing seeds, and PEG 6000-treated leaves, whereas *PfTUB* and *PfTUA* were more stable than *Pf18S* in vegetative organs. *PfEF-1α* ranked last in most sample sets and should not be used as a normalization gene for perilla RT-qPCR analyses. Although the three algorithms produced broadly consistent recommendations, NormFinder ranked *PfEF-1α* as the most stable gene in developing seeds (Table 2), in contrast to geNorm and RefFinder. Such differences arise from the distinct mathematical assumptions of the algorithms. geNorm calculates M values from variation in pairwise expression ratios and can be sensitive to co-regulated gene pairs; NormFinder estimates intra- and intergroup variance components and places greater emphasis on systematic differences among groups; and BestKeeper directly uses the SD and CV of raw Cq values. Integrating the stability scores by their geometric mean with RefFinder reduced model-specific bias and provided a more balanced assessment of overall performance.

The discrepancy regarding *PfEF-1α* in developing seeds reflects the distinct statistical frameworks of the algorithms. NormFinder models both intergroup (developmental stages) and intragroup (biological replicates) variance components, weighting them by their contributions to the overall experimental variance. In developing seeds, *PfEF-1α* showed coordinated upregulation across biological replicates within each stage, resulting in low intragroup variance and a low SV, despite marked fluctuations across stages. geNorm, however, calculates M values from pairwise expression ratios and penalizes genes whose patterns deviate from other candidates—which *PfEF-1α* did owing to its stage-dependent variation. RefFinder integrates all three outputs and placed *PfEF-1α* behind *Pf18S* and *PfTUA* in the final ranking. This algorithm-specific behavior underscores the value of multi-algorithm integration and explains why *PfEF-1α*, despite its favorable NormFinder score in developing seeds, remains unsuitable as a universal reference gene for perilla RT-qPCR analyses.

The results provide both practical reference-gene recommendations and an empirical framework for gene-expression quantification in perilla. In the BestKeeper analysis (Table 3), the SD values of candidate genes frequently exceeded 1.0 across the complete sample set, developing seeds, and vegetative organs, with some values reaching 5–10. This pattern illustrates an important quantitative point: when a dataset spans highly heterogeneous materials—including leaves, stems, roots, multiple seed developmental stages, and osmotic-stress treatments—the absolute expression of conventional housekeeping genes inevitably varies. No single gene can remain perfectly constant across such a broad sample pool. Searching for a universal reference gene for all perilla materials would therefore be statistically inappropriate. This observation supports the use of condition-specific two-gene combinations for vegetative organs, developing seeds, and osmotic stress. Such combinations can reduce background variation across samples and provide more robust normalization within a defined physiological context. It is also worth noting that BestKeeper relies on the SD and CV of raw Cq values and is therefore sensitive to extreme outliers. For instance, the exceptionally low expression of *PfEF-1α* in certain seed samples likely contributed to its inflated SD values in the developing-seed group. This inherent limitation further justifies the integrated multi-algorithm strategy adopted in this study, as geNorm and NormFinder are less susceptible to such outliers, and RefFinder effectively balances the outputs of all three algorithms. Previous RT-qPCR studies of perilla lipid-related genes have generally relied on conventional reference genes without systematic validation. The strategies proposed here for ZS6 tissues, developmental stages, and osmotic-stress conditions provide a methodological basis for subsequent relative-quantification studies and for detailed analysis of the perilla lipid-metabolism regulatory network.

Reference-gene stability in plants is strongly species specific, although the instability of some conventional housekeeping genes appears to be conserved across species. Perilla is an allotetraploid that has undergone polyploidization and contains numerous homologous gene copies, including *PfFAD2* and *PfFAD3* [20]. Transcriptional divergence and regulatory complexity among these copies may influence the background stability of candidate reference genes. Chia (*Salvia hispanica*) is also an ALA-rich oilseed crop, but it differs markedly from perilla in the copy number and structure of its ω-3 fatty-acid desaturase gene family [8]. These genomic differences indicate that optimal reference-gene combinations cannot be extrapolated directly from related species and must be independently evaluated in the relevant genetic and physiological context. Conversely, the poor stability of some conventional housekeeping genes in heterogeneous samples is observed across species. *PfEF-1α* ranked among the least stable genes in most sample sets in the present study, consistent with the highly unstable expression of *NcEF-1α* among tissues of the woody species *Neocinnamomum caudatum* [18]. This cross-species pattern reinforces a central principle of plant RT-qPCR normalization: neither recommendations from other species nor conventional housekeeping genes should be adopted without preliminary, system-specific validation.

In vegetative organs, *PfTUB* and *PfTUA* were consistently ranked among the most stable genes by geNorm, NormFinder, and BestKeeper, and they occupied the top two positions in the integrated RefFinder ranking. Although both genes belong to the tubulin family and encode β- and α-tubulin, respectively [21,22], their geNorm V2/3 value was below the recommended threshold of 0.15 across the vegetative-organ gradient used in this study, indicating that the pair provided sufficient normalization stability. From a strictly data-driven perspective, *PfTUA* + *PfTUB* is therefore the preferred combination for vegetative organs. However, to minimize the potential influence of co-regulation between members of the same gene family, *PfTUB* (ranked first) may alternatively be combined with *Pf18S* (ranked third) when functional independence between reference genes is prioritized.

Differences in reference-gene stability ultimately reflect interactions among gene function, transcriptional regulation, and the specific metabolic context. *Pf18S*, the most broadly stable gene identified here, encodes a GAR2-like 18S pre-ribosomal assembly protein that participates in pre-ribosome assembly and maintenance of the basal translational machinery [23]. Because ribosome biogenesis is a constitutive process required for fundamental cellular activity in plants [24], *Pf18S* transcript levels may remain comparatively stable across developmental stages and stress conditions. By contrast, *PfTUA* and *PfTUB* function in dynamic cytoskeletal remodeling [21,22]. *PfTUA* performed well during seed development, whereas *PfTUB* was more stable in vegetative organs, consistent with tissue-specific roles in cell division and morphogenesis. Acquisition of desiccation tolerance during perilla seed development involves coordinated regulation of lipid metabolism and ABA signaling [25], processes that maintain a sustained demand for the basal translational and ribosome-assembly machinery and may contribute to the robust expression of *Pf18S*. When *Pf18S* was used for normalization, *PfDGAT*, *PfPDAT*, and *PfFAD3* reached expression maxima at SIII (19 days after flowering), qualitatively matching the rapid oil-accumulation period from SII to SIV. This result supports the use of *Pf18S* for capturing broad expression trends, although condition-specific two-gene combinations remain preferable for precise relative quantification. Perilla lipid synthesis is controlled by a multilayered network of transcription factors, including *PfWRI1-10*, *PfDof29*, and *PfbZIP85* [12,13,14]. Accurate relative quantification is essential for resolving the hierarchical relationships in this network, and the reference-gene dataset established here provides a reliable basis for such analyses under the tested conditions.

On the basis of the algorithmic evaluation and physiological validation, we propose a tiered normalization strategy for different research objectives in perilla. For high-throughput preliminary screening and exploration of broad expression trends across tissues or developmental stages, *Pf18S* may be used as a representative single reference gene because it showed the highest integrated stability in most tested sample sets and produced expression patterns consistent with oil and ALA accumulation. This approach can reduce experimental cost and workload while retaining the major biological trends. For precise quantification of small expression differences, hierarchical analysis of transcriptional regulatory networks, or detailed functional validation, condition-specific two-gene combinations should be used: *PfTUA* + *PfTUB* for vegetative organs, *Pf18S* + *PfTUA* for developing seeds, and *PfTUB* + *Pf18S* for osmotic stress. These combinations reduce background noise associated with any single gene and maximize quantitative reliability. The present evaluation covered vegetative organs, five key seed developmental stages, and osmotic stress, but the recommendations remain condition dependent [17,18,19]. Moreover, the biological validation used *Pf18S* alone and did not directly compare normalization with the recommended two-gene combination or with the unstable gene *PfEF-1α*. The agreement between expression patterns and oil accumulation was therefore qualitative rather than a definitive mathematical validation of the two-gene strategy. Future studies should extend the analysis to a wider range of perilla germplasm, larger candidate-gene pools, and additional stresses such as low temperature, salinity–alkalinity, and pathogens. Such work will support the development of a comprehensive, multi-condition reference-gene database for perilla and facilitate epigenetic, multi-omics, and seed-desiccation-tolerance research [6,25,26,27].

## 4. Materials and Methods

### 4.1. Plant Material and Tissue Sampling

The local white perilla accession ZS6 from Gansu Province, China, was used in this study. Surface-sterilized seeds were sown in pots and grown in a greenhouse at 25 ± 2 °C, 70% relative humidity, and a 16 h/8 h light/dark photoperiod. At 30 days after sowing, fully expanded mature leaves, stems, and roots were collected from three independent plants as three biological replicates. Samples were immediately frozen in liquid nitrogen and stored at −80 °C. Seed developmental stages were defined according to days after flowering (DAF): SI, 6 DAF; SII, 13 DAF; SIII, 19 DAF; SIV, 25 DAF; and SV, 31 DAF. To simulate osmotic stress, mature leaves were excised from 30-day-old plants, and the cut ends of the petioles were immediately immersed in 20% (*w*/*v*) PEG 6000. Leaves were collected at 0, 3, 6, 9, 12, and 24 h under conditions of 25 °C, 70% relative humidity, and a light intensity of 100 μmol m^−2^ s^−1^, immediately frozen in liquid nitrogen, and used for RNA extraction. Three independent biological replicates were collected for each sample, and each replicate comprised pooled tissues from three independent plants.

### 4.2. Total RNA Extraction and cDNA Synthesis

Total RNA was extracted from the tissues and treatment samples using a Plant RNA Rapid Extraction Kit (RN0102; Aidlab, Beijing, China). Residual genomic DNA was removed by treatment with DNase I (RQ1; Promega, Madison, WI, USA). RNA integrity was assessed by 1% agarose-gel electrophoresis, and samples with clear, non-degraded bands were retained (Supplementary Figure S1). RNA concentration and purity were measured using a NanoDrop ND-2000 spectrophotometer (Thermo Fisher Scientific, Waltham, MA, USA), and A260/A280 ratios ranged from 1.8 to 2.1. First-strand cDNA was synthesized from 1 μg of total RNA using the PrimeScript II First Strand cDNA Synthesis Kit (6210A; Takara, Beijing, China) and stored at −20 °C until use.

### 4.3. Selection of Candidate Reference Genes and Primer Design

Transcriptome sequencing was performed on pooled samples of leaves, stems, and seeds at SI, SIII, and SV from ZS6. After quality control, Trinity assembly, and expression quantification, fragments per kilobase of transcript per million mapped reads (FPKM) values were obtained. KEGG enrichment analysis showed that differentially expressed genes were significantly enriched in 20 major metabolic pathways, including ribosome and fatty-acid biosynthesis (Supplementary Figure S2A). A Venn analysis of differentially expressed genes across tissues and developmental stages identified 144 core co-expressed genes (Supplementary Figure S2B). Sequences of conventional housekeeping genes reported in *Arabidopsis thaliana* were used as queries for local BLAST searches with BioEdit v7.0.9.0. Seven candidate reference genes were initially selected: *PfActin*, *Pf18S*, *PfEF-1α*, *PfCYP*, *PfTUA*, *PfTUB*, and *PfGAPDH*. Their nucleotide and protein sequences are provided in Supplementary Material File S1. Expression abundance and stability were examined in the transcriptome dataset and across the RT-qPCR Cq-value distribution. *PfGAPDH* was excluded because of its high median Cq value and highly variable expression among treatments. Gene-specific primers were designed within the open reading frames using Vector NTI Advanced v11.5 or Primer Premier 5.0. Primer sequences, amplicon lengths, and GenBank accession numbers are listed in Supplementary Table S1. Primer lengths were 18–22 bp, GC contents were 40–60%, melting temperatures were approximately 60 °C, and amplicon sizes were 150–220 bp.

### 4.4. RT-qPCR and Determination of Amplification Efficiency

RT-qPCR was performed using a Bio-Rad CFX96 Real-Time PCR Detection System (Bio-Rad, Hercules, CA, USA) and TB Green Premix Ex Taq II (RR820A; Takara, Beijing, China). Each 20 μL reaction contained 10 μL of SYBR Premix, 1 μL of cDNA template, 0.5 μL of each forward and reverse primer (10 μM), and 8 μL of ddH2O. The amplification program consisted of 3 min at 95 °C, followed by 40 cycles of 95 °C for 10 s and 58 °C for 30 s. Melting curves were generated from 65 to 95 °C with fluorescence acquisition at 0.5 °C increments to assess amplification specificity. To determine amplification efficiency, pooled cDNA was serially diluted fivefold (1, 1/5, 1/25, 1/125, 1/625, and 1/3125), with three technical replicates per dilution. Standard curves were generated by plotting Cq against the logarithm of the dilution factor, and R^2^ and slope values were calculated. Amplification efficiency was calculated as E (%) = [10^(−1/slope) − 1] × 100. Primer pairs were retained only when R^2^ > 0.98 and E was between 90% and 110%. Raw data for the standard curves and dilution series are provided in Supplementary Material Table S3.

### 4.5. Evaluation of Reference-Gene Stability and Multi-Algorithm Integration

Raw Cq values obtained for the six candidate reference genes across tissues, seed developmental stages, and stress treatments were analyzed using geNorm, NormFinder, and BestKeeper. geNorm analysis was performed using the geNorm Excel macro (version 3.5; available at https://genorm.cmgg.be/). NormFinder analysis was conducted using the NormFinder Excel macro (https://moma.dk/normfinder-software/ (accessed on 19 July 2026)). BestKeeper analysis was carried out using the BestKeeper Excel-based tool (version 1; available at http://bioinformatics.gene-quantification.info/bestkeeper.html (accessed on 19 July 2026)). RefFinder is available at https://blooge.cn/RefFinder/ (accessed on 19 July 2026), and the version used in this study was accessed in April 2025. geNorm ranks genes by their average expression-stability value (M; lower values indicate greater stability) and calculates pairwise variation (V_n_/V_n+1_) to determine the optimal number of reference genes, using 0.15 as a conventional threshold. NormFinder estimates inter- and intragroup variation using a model-based approach and reports a stability value (SV; lower values indicate greater stability). BestKeeper ranks genes directly from the SD and CV of the raw Cq values. To reduce potential bias from any single algorithm, the outputs were integrated using the RefFinder online tool (https://blooge.cn/RefFinder/ (accessed on 19 July 2026)), which generated a final geometric-mean stability score for each sample set. The raw Cq matrices and complete recalculated outputs are provided in Supplementary Material Table S4.

### 4.6. Validation Using Target-Gene Expression Patterns

To evaluate the applicability of the selected reference gene in studies of perilla lipid metabolism, *Pf18S*, the top-ranked single reference gene, was used to normalize the spatiotemporal expression of six lipid-biosynthesis-related genes (*PfDGAT*, *PfPDAT*, *PfFAD2*, *PfFAD3*, *PfLPCAT1.1*, and *PfLPCAT1.2*) in leaves, stems, roots, and developing seeds. Relative expression was calculated using the 2^(−ΔΔCt) method. Target-gene Cq values were first normalized to *Pf18S* to account for variation in RNA input and reverse-transcription efficiency, and expression in stems was used as the calibrator and set to 1.0. Each experiment included three biological replicates and three technical replicates. Data are presented as the mean ± standard error.

### 4.7. Determination of Seed Oil Content and Gas-Chromatographic Analysis

Total lipid extraction, fatty-acid methylation, and gas-chromatographic analysis of seeds at different developmental stages were performed using a previously reported method with minor modifications. Briefly, 50 mg of seed material was accurately weighed and ground. A 500 μL aliquot of hexane containing 0.5 mg mL^−1^ triheptadecanoin (C17:0 TAG) as the quantitative internal standard was added, and the sample was homogenized at high speed for 2 min. After centrifugation at 3000 rpm for 8 min at 4 °C, 100 μL of the clear supernatant was transferred to a 5 mL pressure-resistant glass tube. Two milliliters of freshly prepared 1% (*v*/*v*) concentrated sulfuric acid in methanol was added, the tube was tightly capped and mixed, and methylation was performed in an 80 °C water bath for 2 h. After cooling to room temperature, 2 mL of 0.9% (*w*/*v*) NaCl was added to terminate the reaction, followed by 2 mL of hexane. The mixture was vigorously extracted for 2 min and centrifuged at 2000 rpm for 3 min. The upper hexane phase was collected, and the extraction was repeated once. The combined hexane fractions were evaporated under a gentle stream of nitrogen, and the fatty-acid methyl esters (FAMEs) were redissolved in 100 μL of hexane and transferred to autosampler vials fitted with inserts. Gas chromatography was performed using an Agilent 7890B system (Agilent Technologies, Santa Clara, CA, USA) equipped with a flame-ionization detector and a DB-23 fused-silica capillary column (60 m × 0.32 mm internal diameter, 0.25 μm film thickness, Agilent Technologies, Santa Clara, CA, USA). The injector temperature was 250 °C; samples (1 μL) were injected in split mode at a split ratio of 20:1. The detector temperature was 280 °C, and high-purity helium was used as the carrier gas at 1.5 mL min^−1^. The oven program was 150 °C for 1 min, increased to 210 °C at 4 °C min^−1^ and held for 5 min, then increased to 230 °C at 10 °C min^−1^ and held for 5 min. A 37-component FAME standard mixture (Supelco 37 Component FAME Mix, Supelco, Bellefonte, PA, USA) was analyzed in parallel. Fatty acids were identified by retention time, and the C17:0 methyl-ester peak was used as the quantitative internal standard. Peak-area integration was used to calculate the absolute amount and percentage of each fatty acid and to estimate total seed oil content. The underlying data are provided in Supplementary Material Table S2.

## 5. Conclusions

Seven conventional housekeeping genes were evaluated as candidate reference genes for RT-qPCR in *Perilla frutescens*. After *PfGAPDH* was excluded because of low abundance and unstable expression, the remaining six genes were systematically assessed in vegetative organs, developing seeds, and PEG 6000-treated leaves. Integrated analysis using geNorm, NormFinder, BestKeeper, and RefFinder identified *Pf18S* as the most stable gene in the complete sample set, developing seeds, and osmotic-stress samples, whereas *PfTUB* and *PfTUA* were preferred in vegetative organs. The recommended two-gene combinations are *PfTUA* + *PfTUB* for vegetative organs, *Pf18S* + *PfTUA* for developing seeds, and *PfTUB* + *Pf18S* for osmotic stress. These validated normalization strategies provide a methodological foundation for accurate gene-expression analysis and for elucidating the molecular regulation of lipid metabolism in perilla.

## Figures and Tables

**Table 1:** RT-qPCR standard curves and amplification efficiencies of the candidate reference genes.

Gene	Description	GenBank Accession Number	Standard Curve	Slope	R^2^	PCR Efficiency (%)
*PfActin*	Actin	PZ587224	y = −3.4750x + 31.180	−3.4750	0.9983	93.99
*Pf18S*	18S pre-ribosomal assembly protein GAR2-like	PZ587225	y = −3.3756x + 31.072	−3.3756	0.9958	97.81
*PfEF-1α*	Elongation factor 1-alpha	PZ587226	y = −3.5011x + 37.846	−3.5011	0.9995	93.03
*PfCYP*	Cyclophilin	PZ587228	y = −3.4504x + 35.448	−3.4504	0.9902	94.90
*PfTUA*	Alpha-tubulin	PZ587229	y = −3.4573x + 35.458	−3.4573	0.9900	94.64
*PfTUB*	Beta-tubulin	PZ587230	y = −3.2873x + 34.664	−3.2873	0.9977	101.47
*GAPDH*	Glyceraldehyde-3-phosphate dehydrogenase	PZ587227	y = −3.5518x + 39.488	−3.5518	0.9932	91.22

**Table 2:** NormFinder stability values of candidate reference genes in the different sample sets.

All Samples		Developing Seeds		Leaf & Stem & Root		PEG 6000 Treatment	
Reference Gene	Stability Value	Reference Gene	Stability Value	Reference Gene	Stability Value	Reference Gene	Stability Value
*Pf18S*	0.715	*PfEF-1α*	0.147	*PfTUB*	0.328	*PfTUB*	0.316
*PfTUB*	0.804	*Pf18S*	0.186	*PfTUA*	0.868	*PfActin*	0.344
*PfActin*	1.292	*PfTUA*	0.421	*Pf18S*	1.194	*Pf18S*	0.392
*PfCYP*	1.434	*PfActin*	0.533	*PfCYP*	1.208	*PfCYP*	0.801
*PfTUA*	1.482	*PfCYP*	0.763	*PfActin*	1.868	*PfTUA*	0.825
*PfEF-1α*	1.923	*PfTUB*	0.795	*PfEF-1α*	2.213	*PfEF-1α*	0.949

**Table 3:** BestKeeper stability statistics of candidate reference genes in the different sample sets.

All Samples			Leaf & Stem & Root			Developing Seeds			PEG 6000 Treatment		
*RG*	Std Dev	CV	*RG*	Std Dev	CV	*RG*	Std Dev	CV	*RG*	Std Dev	CV
*PfCYP*	5.84	2.61	*PfTUA*	2.16	1.39	*Pf18S*	2.75	1.53	*Pf18S*	0.32	1.2
*Pf18S*	6.96	2.72	*Pf18S*	2.64	1.42	*PfTUB*	2.60	1.65	*PfTUB*	0.32	1.32
*PfEF-1α*	6.03	3.04	*PfTUB*	2.35	1.47	*PfTUA*	2.76	1.68	*PfTUA*	0.54	2.23
*PfTUB*	7.67	3.91	*PfCYP*	5.79	2.54	*PfActin*	3.01	1.70	*PfCYP*	0.58	2.44
*PfActin*	8.49	4.07	*PfActin*	8.43	3.66	*PfEF-1α*	3.07	1.78	*PfEF-1α*	0.96	3.66
*PfTUA*	10.26	5.76	*PfEF-1α*	8.45	4.64	*PfCYP*	5.55	2.54	*PfActin*	1.12	5.09

Note: SV, stability value; RG, reference gene; SD, standard deviation; CV, coefficient of variation. Values were calculated from Cq measurements obtained from three biological replicates.

## Data Availability

The sequence data for the seven candidate reference genes have been submitted to GenBank under the submission ID SUB16281403 with the following accession numbers: *PfActin*: PZ587224, *Pf18S*: PZ587225, *PfEF-1α*: PZ587226, *PfGAPDH*: PZ587227, *PfCYP*: PZ587228, *PfTUA*: PZ587229, *PfTUB*: PZ587230.

## Supplementary Materials

The following supporting information can be downloaded at: https://www.mdpi.com/article/10.3390/plants15172588/s1. The following supporting information accompanies this manuscript: Figure S1, RNA integrity assessment; Figure S2, transcriptome pathway enrichment, co-expression analysis, and lipid-metabolism heatmap. (**A**) KEGG pathway enrichment dot plot of the top 20 enriched metabolic pathways. Bubble size represents the number of genes, and color intensity indicates the P-value significance. (**B**) Venn diagram of differentially expressed genes (DEGs) across different tissue and seed development stage comparisons (LEAF, STEM, SEED1–SEED3). The central intersection highlights 144 core shared DEGs. (**C**) Schematic representation of the lipid biosynthesis and TAG assembly pathways in the plastid and endoplasmic reticulum (ER), along with expression heatmaps (Log2 FPKM) of key genes and transcription factors; Table S1, primer sequences and amplicon information; Table S2, seed oil and fatty-acid data; Table S3, standard curves and raw dilution-series Cq values; Table S4, raw Cq matrices and stability-algorithm outputs; File S1, nucleotide and protein sequences of candidate reference genes.
