# Supplementary material for: Selection and Validation of Reference Genes for RT-qPCR Analysis of Lipid Biosynthesis-Related Genes in Perilla frutescens
Source: Plants (Basel). 2026 Aug 25;15(17):2588. doi: 10.3390/plants15172588 (PMC13567130; doi:10.3390/plants15172588)
Supplement: Supplementary file 1 [file plants-15-02588-s001.zip › Figure S1.pdf]

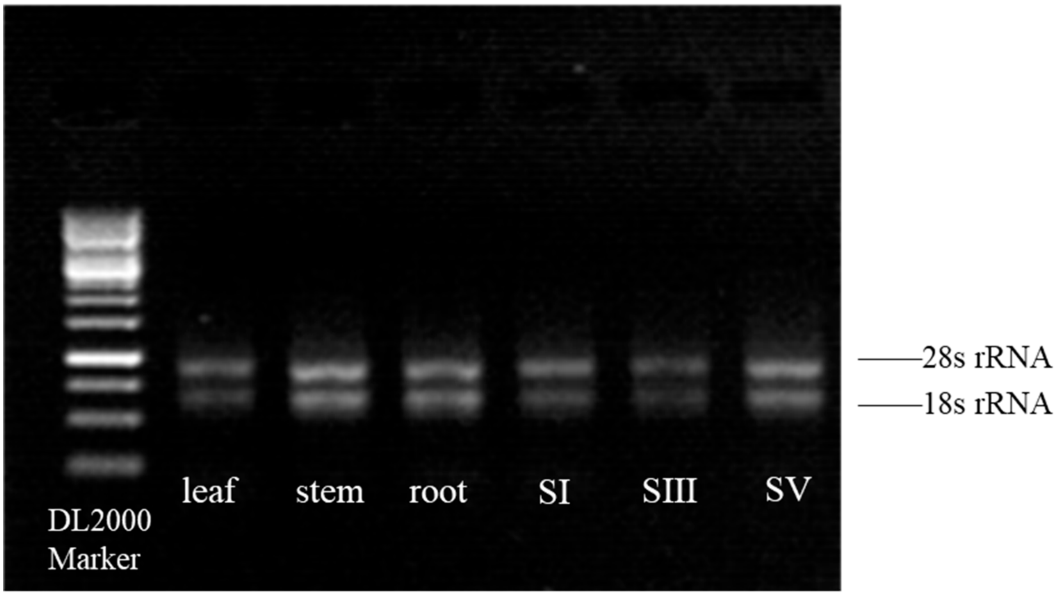

Figure S1, RNA integrity assessment
