# Supplementary material for: Selection and Validation of Reference Genes for RT-qPCR Analysis of Lipid Biosynthesis-Related Genes in Perilla frutescens
Source: Plants (Basel). 2026 Aug 25;15(17):2588. doi: 10.3390/plants15172588 (PMC13567130; doi:10.3390/plants15172588)
Supplement: Supplementary file 1 [file plants-15-02588-s001.zip › Figure S2.pdf]

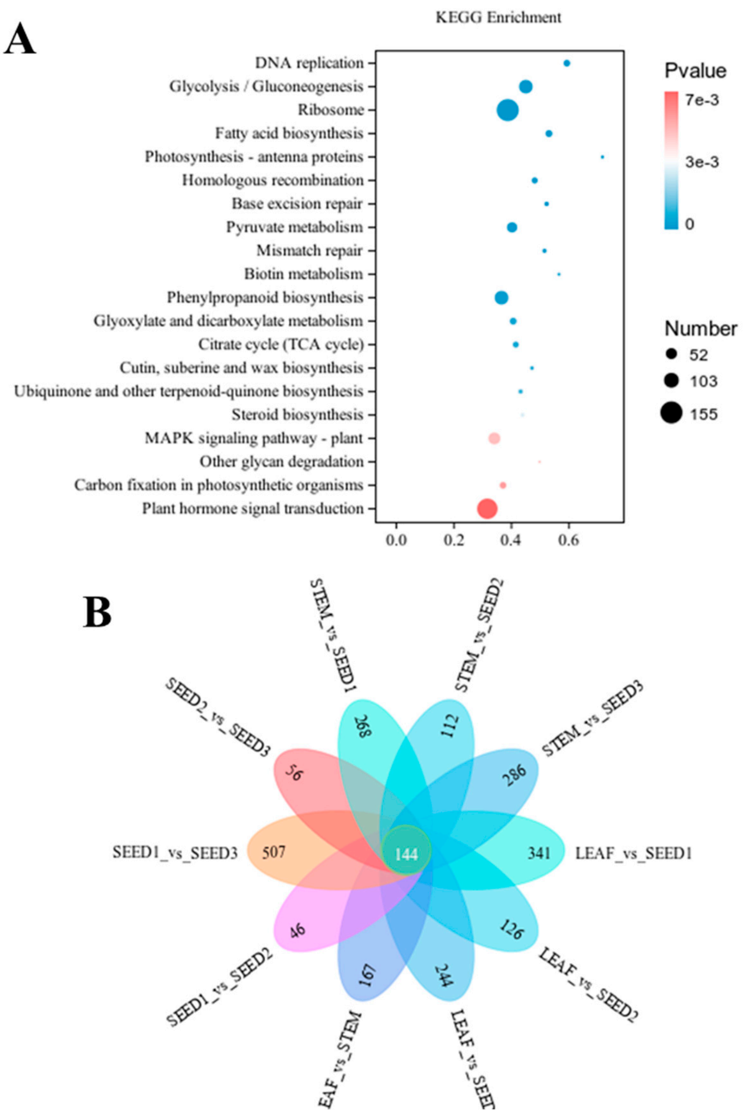

Figure S2, transcriptome pathway enrichment, co-expression analysis, and lipid-metabolism heatmap (A) KEGG pathway enrichment dot plot of the top 20 enriched metabolic pathways. Bubble size represents the number of genes, and color intensity indicates the P-value significance. (B) Venn diagram of differentially expressed genes (DEGs) across different tissue and seed development stage comparisons (LEAF, STEM, SEED1–SEED3). The central intersection highlights 144 core shared DEGs.

C

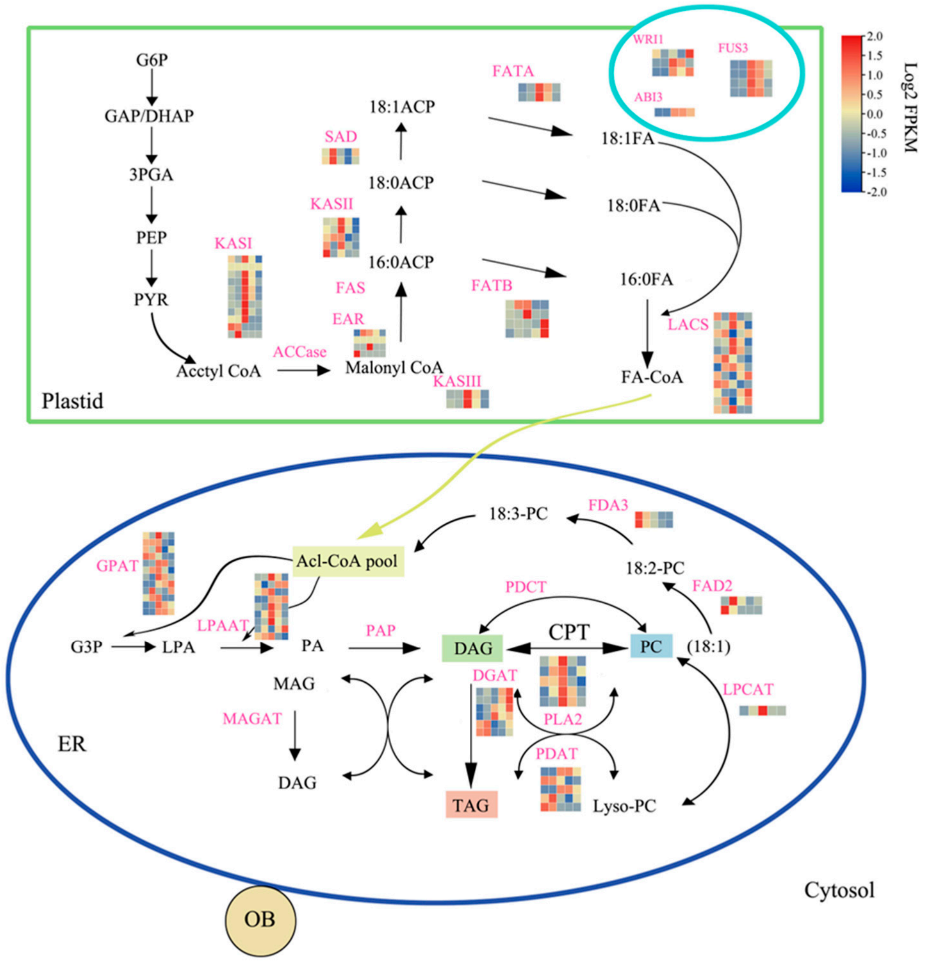

Figure S2 C Schematic representation of the lipid biosynthesis and TAG assembly pathways in the plastid and endoplasmic reticulum (ER), along with expression heatmaps (Log2 FPKM) of key genes and transcription factors.
