# Supplementary material for: Selection and Validation of Reference Genes for RT-qPCR Analysis of Lipid Biosynthesis-Related Genes in Perilla frutescens
Source: Plants (Basel). 2026 Aug 25;15(17):2588. doi: 10.3390/plants15172588 (PMC13567130; doi:10.3390/plants15172588)
Supplement: Supplementary file 1 [file plants-15-02588-s001.zip › File S1.pdf]

File S1, nucleotide and protein sequences of candidate reference genes

Actin：

>KAH6806310.1 actin-11 [Perilla frutescens var. frutescens]

Length = 377 Score = 740 bits (1910), Expect = 0.0

Identities = 364/377 (96%), Positives = 372/377 (98%)

MAEAEDIQPLVCDNGTGMVKAGFAGDDAPRAVFPSIVGRPRHTG

VMVGMGQKDAYVGDEAQSKRGILTLKYPIEHGIVSNWDDMEKI

WHHTFYNELRVAPEEHPVLLTEAPLNPKANREKMTQIMFETFNA

PAMYVAIQAVLSLYASGRTTGIVLDSGDGVSHTVPIYEGYSLPHAI

LRLDLAGRDLTDYLMKILTERGYSFTTSAEREIVRDMKEKLSYIA

LDYEQELETAKTSSSVEKSYELPDGQVITIGNERFRCPEVLFQPSMI

GMEAAGVHETTYNSIMKCDVDIRKDLYGNIVLSGGTTMFTGIAD

RMSKEITALAPSSMKIKVVAPPERKYSVWIGGSILASLSTFQQMWI

AKAEYDESGPSIVHRKCF

>lcl|QFCC02000007.1_cds_KAH6806310.1_35078

[locus_tag=C2S51_031141] [protein=actin-11]

[protein_id=KAH6806310.1]

[location=join(45482903..45482962,45483061..45483454,45483778..454

84391,45484481..45484546)] [gbkey=CDS]

ATGGCCGAAGCAGAGGATATTCAGCCACTCGTCTGCGACAATG

GAACTGGAATGGTTAAGGCTGGATTTGCTGGAGATGATGCTCC

CCGAGCTGTGTTTCCTAGCATTGTTGGACGGCCCCGTCACACA

GGTGTGATGGTGGGTATGGGCCAAAAAGATGCATATGTTGGTG

ATGAAGCTCAGTCAAAGAGAGGTATTTTAACTCTGAAGTACCC

TATTGAGCATGGTATTGTCAGCAACTGGGATGATATGGAGAAG

ATTTGGCATCACACGTTCTACAATGAGCTACGTGTTGCCCCGG

AAGAGCACCCTGTTCTCCTGACTGAGGCACCACTCAACCCTAA

GGCTAACAGGGAGAAGATGACTCAGATTATGTTCGAGACATTC

AATGCTCCTGCTATGTACGTTGCCATTCAGGCCGTCCTTTCCCT

GTATGCCAGTGGTCGTACTACTGGTATTGTGCTGGATTCTGGTG

ATGGTGTCAGTCACACTGTCCCAATCTACGAGGGGTATTCCCT

CCCACATGCCATCCTTCGTCTTGACTTGGCTGGCCGTGACCTTA

CTGATTACTTGATGAAGATTCTTACCGAGCGTGGCTACTCTTTC

ACCACCTCTGCAGAGCGGGAAATTGTGAGAGACATGAAAGAA

AAGTTATCTTACATTGCCCTTGACTACGAGCAGGAGCTGGAGA

CTGCAAAGACTAGTTCTTCCGTGGAGAAGAGCTACGAGTTGCC

TGACGGGCAGGTGATTACCATTGGTAATGAGCGTTTCCGTTGC

CCTGAGGTCCTCTTCCAGCCGTCAATGATAGGTATGGAAGCAG

CTGGAGTCCACGAGACCACGTACAACTCCATCATGAAGTGCGA

CGTGGATATTAGGAAGGACTTGTATGGTAACATCGTACTCAGT

GGTGGTACGACCATGTTCACTGGCATTGCCGACAGAATGAGCA

AGGAAATCACAGCCCTTGCTCCGAGCAGCATGAAGATCAAGG

TCGTTGCTCCTCCTGAGAGGAAATACAGTGTCTGGATCGGAGG

CTCGATCTTGGCCTCTCTCAGCACTTTCCAACAGATGTGGATTG

CAAAGGCTGAATATGATGAGTCTGGTCCATCTATCGTGCACAG
GAAATGCTTCTAA

18S:

>KAH6802157.1 18S pre-ribosomal assembly protein gar2-like protein[Perilla frutescens var. frutescens]

Length = 497 Score = 92.3 bits (203), Expect(3) = 1e-033

Identities = 40/57 (70%), Positives = 47/57 (82%)

MLAAQLLRVLQTIPSDVNHETCVYDSTAQDMIMKENQNGTNHQ
DEPFRRVDSQEDDKDSLSSDTSTKDMVQTRPEHDFPVEGRTFIHC
NENGSRDSRSSYVAASDLYTDKNVLEYERPELIVCYKEISYHVVK
DICMDEGKPANRNFFIDSSKVDQSGHSFPQPLNDDNHCKATGAV
DEELSISNGLAATSLENAEFGFASQQGSKKERNVILPSERSLYKDV
SMGCNLGESKQEGGAIIGATSKTAMHASNEESFVGRTLPIQEFGT
RSFLRSFLNSLDDEEDKVEQLPDQFSSGGVVSEGPPATLKKAEPKE
DVRATILCYNSEVETRSITFNFNSPTPVVGGITNGKSELVKERSDSR
EVIDQKDAKVDIFSYSRQDQACSVGNGNMKTDDEEDMFAESNIL
QHKDSGSVVSLRKYDQGESSFSADDFIVHSGPIAFSGSLSLCSDGS
ATSGRSFAFPILQSEWNNSPVRMTEAERRRFRKHKGWRSGLLCCR
F

ATGTTGGCTGCACAACTGCTGAGGGTTCTCCAAACTATCCCTA
GTGATGTGAATCATGAAACCTGCGTCTATGACTCGACTGCTCA
AGACATGATCATGAAGGAAAATCAGAACGGAACGAATCATCA

AGATGAACCTTTTCGTCGTGTGGATAGTCAGGAGGATGATAAA

GATTCATTATCTTCTGACACAAGTACTAAAGACATGGTGCAAA

CCAGGCCAGAGCATGATTTTCCTGTCGAGGGTAGAACGTTTAT

CCATTGTAATGAAAATGGATCAAGAGATAGTAGATCTTCATAT

GTAGCTGCTTCAGACTTGTATACAGATAAAAATGTATTGGAAT

ATGAACGGCCTGAGTTAATAGTTTGCTATAAGGAGATCAGTTA

TCATGTTGTAAAGGATATATGCATGGATGAGGGAAAGCCTGCA

AACAGAAATTTTTTTATTGATAGTAGCAAGGTTGACCAGTCTG

GCCATTCGTTTCCCCAACCTCTAAATGATGATAACCATTGTAA

AGCTACCGGAGCTGTTGACGAGGAACTTTCCATATCAAATGGA

TTAGCAGCTACATCACTGGAAAATGCAGAGTTTGGTTTTGCTA

GTCAGCAGGGGAGCAAAAAAGAAAGAAATGTGATATTGCCCT

CAGAAAGATCTTTGTACAAGGATGTTTCCATGGGTTGTAATCT

GGGGGAGTCGAAGCAAGAAGGTGGGGCAATCATTGGTGCAAC

CAGCAAAACTGCAATGCATGCTTCAAATGAGGAGTCTTTTGTA

GGCAGGACTCTTCCAATTCAAGAATTCGGCACGCGAAGTTTCC

TCAGATCTTTTCTGAATTCTTTAGATGATGAGGAAGATAAAGT

CGAACAGCTGCCAGATCAGTTTTCGTCTGGAGGAGTGGTTTCG

GAAGGTCCTCCTGCAACGTTAAAAAAAGCAGAGCCAAAGGAA

GACGTGCGAGCAACCATCTTATGTTACAACAGTGAGGTGGAAA

CAAGAAGCATAACATTCAATTTCAACTCCCCGACACCCGTCGT

AGGTGGCATTACAAATGGGAAAAGTGAACTCGTTAAGGAACG

GTCTGACTCAAGAGAGGTGATCGACCAAAAAGACGCAAAAGT

TGACATTTTTTCATATTCTAGGCAAGATCAAGCTTGTAGTGTTG

GTAACGGCAATATGAAGACTGATGACGAGGAAGATATGTTTGC

AGAGTCAAATATTTTGCAGCATAAAGATAGTGGTTCAGTTGTT

AGCTTACGGAAGTATGACCAGGGGGAGTCGAGCTTTTCTGCAG

ACGATTTTATTGTGCACTCGGGGCCAATAGCATTTTCAGGAAG

CCTCTCCCTTTGCTCGGATGGCAGTGCAACCAGTGGCAGATCT

TTCGCTTTCCCAATATTACAATCGGAATGGAATAACAGTCCAG

TGAGAATGACAGAAGCAGAAAGGAGACGTTTTCGGAAACATA

AGGGTTGGAGATCAGGCCTTCTCTGTTGTAGATTCTAA

EF-1α

>KAH6806780.1 Translation elongation factor EF1A/initiation factor IF2gamma family protein [Perilla frutescens var. frutescens]

Length = 678 Score = 692 bits (1785), Expect = 0.0

Identities = 372/674 (55%), Positives = 465/674 (68%), Gaps = 54/674 (8%)

MATKTMPAKEASTTTAVWRCPICTYDNEDSMSACDICGVLRNPL

VKTNIKTDNIAVGGMCKDSGASVMAKSLFASLPQRMAKASILEV

QNDVPSTDNHVNFHKLGNMHGTFPESSSAFSYQNHKKVTIAPFKF

DAPSPDDLVSSGLQSRKLKSKGSERDTSVPVELLLAAKEQESTSTS

VTRRRSNGVGEKNDTPSRGNKPEMSVSNVNTEALSSKTGKAEITR

ERKTASISEFTPETWMIPDTVEGELSQLNLAIVGHVDSGKSTLSGR
LLHLSGRISQKEMHKYEKEAKQQGKGSFAYAWALDESAEERERG
ITMTVGVAFFTSRKYHVVLLDSPGHRDFVPNMISGATQADAAILV
VDASMGSFEAGIDATGGQTREHVQLIRSFGVDQIIVLVNKMDVVE
YSKERFDFVKQKLGAFLRTCGFKESSISWVPASAMENQNLVAKSS
DARLSWYKGPSLMDGIDSLQLPARDYSKPLLLPICDVKSQSQSQV
SACGKLESGAIRSGFKVLVMPSKEIATARSLERDSQPCSIARAGDN
VTVNLQGTEGNRVAAGSVICHPDYPVQVASQLELKILVLEISTPIV
IGSQLEFHIHHAKEAAKIVKILSLLDPKTGKVTKKSPRCLLAKQNA
MVEVALQGPVCVEEYSSCRALGRVFLRASGRTIALGLVTRIIQRE
N

ATGGCCACTAAGACGATGCCTGCAAAAGAAGCCAGCACCACA
ACTGCGGTCTGGCGATGCCCAATTTGTACCTATGATAATGAAG
ATAGTATGTCTGCCTGTGATATATGTGGGGTTTTACGCAACCCT
TTGGTCAAGACTAATATTAAAACCGATAATATTGCAGTGGGTG
GCATGTGCAAAGATTCTGGAGCATCTGTAATGGCCAAGTCTCT
TTTTGCATCATTGCCGCAACGTATGGCCAAAGCATCCATCCTTG
AAGTGCAGAATGACGTTCCTTCCACAGATAACCACGTTAACTT
CCACAAACTCGGAAATATGCATGGCACTTTTCCAGAATCCAGT
AGTGCTTTTAGTTACCAAAATCATAAAAAAGTTACTATAGCCC
CTTTCAAATTCGATGCTCCTTCTCCAGATGATTTGGTGTCAAGT
GGACTGCAATCACGGAAATTGAAATCTAAAGGCAGTGAGAGG

GACACAAGTGTTCCAGTTGAACTGTTGTTGGCTGCTAAAGAGC

AAGAGTCCACTTCGACATCAGTAACAAGAAGGAGAAGTAATG

GAGTTGGTGAGAAAAATGACACTCCATCCCGTGGAAACAAAC

CTGAGATGTCTGTTAGCAATGTGAACACTGAGGCTCTGTCTTC

CAAAACTGGAAAAGCAGAAATAACTCGTGAGAGAAAAACGGC

ATCAATTTCTGAGTTCACTCCTGAGACATGGATGATTCCAGAT

ACAGTTGAGGGTGAATTGAGCCAGCTAAATCTTGCAATCGTTG

GCCATGTTGATTCTGGGAAGTCAACACTTTCAGGAAGGTTGCT

TCATCTATCGGGACGAATATCTCAGAAAGAGATGCACAAATAC

GAAAAAGAAGCTAAACAACAGGGAAAAGGATCTTTTGCTTAT

GCATGGGCATTGGATGAAAGTGCTGAAGAAAGAGAAAGGGGT

ATAACTATGACGGTGGGCGTTGCGTTTTTACTTCTAGAAAATA

TCACGTTGTGTTGCTTGACTCCCCTGGACATAGAGATTTTGTTC

CGAACATGATCTCTGGAGCAACACAAGCAGATGCTGCAATTTT

GGTGGTTGATGCTTCAATGGGTTCATTTGAAGCCGGCATAGAT

GCTACCGGAGGGCAAACGAGGGAACATGTACAGCTAATTAGG

AGCTTTGGAGTTGATCAGATCATTGTTCTTGTCAACAAAATGG

ATGTGGTTGAATACTCAAAGGAAAGATTTGATTTTGTGAAGCA

GAAACTAGGGGCATTTCTCCGTACTTGCGGTTTTAAAGAGTCTT

CTATTTCATGGGTTCCAGCAAGTGCCATGGAAAACCAGAACTT

AGTTGCAAAATCTTCTGATGCACGGTTGTCCTGGTATAAAGGA

CCTTCTTTGATGGATGGAATAGATTCTCTCCAACTTCCTGCAAG

AGATTACTCCAAACCACTACTACTTCCTATATGCGATGTTAAGT

CACAATCCCAAAGTCAGGTGTCTGCATGTGGGAAATTGGAAAG

TGGAGCTATTAGATCAGGATTTAAGGTATTGGTCATGCCTTCA

AAAGAAATAGCAACCGCCCGCTCGTTGGAACGGGACTCCCAG

CCTTGTAGTATTGCGAGAGCTGGTGACAATGTTACAGTCAATT

TGCAAGGTACTGAGGGCAATCGTGTAGCAGCTGGAAGTGTGAT

ATGTCACCCTGACTACCCAGTTCAAGTCGCAAGCCAGTTGGAA

CTGAAAATTCTTGTCCTTGAAATTTCGACCCCGATTGTGATCGG

TTCTCAGTTGGAGTTTCACATACACCACGCAAAAGAGGCTGCA

AAAATTGTGAAGATTTTGTCACTGCTTGATCCAAAGACAGGGA

AAGTGACAAAGAAGTCGCCAAGATGCTTATTGGCCAAGCAGA

ATGCCATGGTTGAGGTGGCTTTGCAAGGTCCCGTTTGCGTGGA

GGAGTACAGTAGTTGCAGAGCTCTGGGAAGAGTGTTTCTTAGA

GCATCAGGAAGGACGATTGCTCTTGGTCTTGTGACTCGAATCA

TTCAGAGGGAAAACTAG

GAPDH

>KAH6773975.1 glyceraldehyde 3-phosphate dehydrogenase A subunit [Perilla frutescens var. frutescens]

Length = 398 Score = 673 bits (1737), Expect = 0.0

Identities = 337/386 (87%), Positives = 362/386 (93%), Gaps = 4/386 (1%)

MASAALSSLQVGCSKGGFSEFAGLRSSSAALPFAKRTNDDFVSLI

AFQTSVVGGGKRGVVEAKLKVAINGFGRIGRNFLRCWHGRKDSP

LDVIAINDTGGVKQASHLLKYDSTLGIFDADVKPVGTDGISVDGK

VIKVVSDRNPANLPWKELGIDLVIEGTGVFVDRDGAGKHITAGAK

KVLITAPGKGDIPTYVVGVNADDYSDDEPIISNASCTTNCLAPFVK

VLDQKFGIIKGTMTTTHSYTGDQRLLDASHRDLRRARAAALNIVP

TSTGAAKAVALVLPQLKGKLNGIALRVPTPNVSVVDLVVQVEKK

TFAEEVNEAFRESASKELNGILSVCDEPLVSVDFRCSDVSSTVDSS

LTMVMGDDMVKVIAWYDNEWGYSQRVVDLADIVASKWK

ATGGCTTCTGCTGCTCTTTCATCTCTGCAGGTGGGCTGCAGCAA

GGGCGGCTTCTCTGAGTTTGCTGGGCTCCGAAGCTCCTCCGCC

GCTCTGCCCTTCGCCAAGAGGACCAACGATGATTTCGTTTCTCT

TATTGCCTTCCAGACCTCTGTTGTTGGTGGAGGCAAGAGGGGA

GTAGTGGAGGCGAAGTTGAAAGTGGCGATCAATGGATTTGGTA

GGATTGGAAGGAACTTCTTGAGGTGCTGGCATGGAAGAAAGG

ACTCCCCTCTCGATGTCATTGCCATTAACGACACCGGAGGTGT

GAAGCAAGCCTCCCACCTCCTCAAATACGACTCCACCCTCGGC

ATCTTCGACGCCGACGTCAAGCCAGTCGGCACCGACGGCATCT

CCGTCGACGGCAAGGTCATCAAAGTCGTCTCCGACCGCAACCC

TGCCAACCTCCCCTGGAAGGAGCTGGGCATCGATCTTGTGATC

GAGGGAACTGGAGTGTTCGTGGACAGAGATGGTGCAGGGAAG

CACATCACAGCAGGGGCGAAGAAAGTGCTCATCACCGCCCCC

GGAAAAGGCGACATTCCGACGTATGTTGTTGGTGTCAATGCAG

ACGACTACAGCGACGACGAGCCCATCATCAGCAACGCTTCCTG

CACCACCAACTGCCTCGCTCCTTTCGTCAAAGTCCTCGACCAG

AAGTTCGGAATCATCAAGGGAACCATGACCACCACACACTCCT

ACACCGGAGACCAGAGGCTGCTCGATGCAAGCCACCGTGACTT

GAGGCGTGCACGGGCTGCAGCGCTCAACATTGTGCCTACATCC

ACCGGTGCTGCAAAGGCTGTGGCCCTCGTCCTTCCCCAGCTCA

AGGGCAAGCTCAATGGCATTGCCCTCCGTGTTCCCACCCCCAA

TGTCTCCGTGGTGGACCTCGTGGTTCAGGTCGAGAAAAAGACT

TTCGCAGAGGAAGTGAATGAGGCTTTCAGGGAGTCCGCCAGC

AAGGAGCTCAACGGCATCTTGTCCGTGTGTGACGAGCCACTCG

TCTCAGTCGACTTCCGTTGCAGCGATGTCTCCTCCACCGTCGAC

TCCTCACTCACTATGGTCATGGGAGATGACATGGTTAAGGTCA

TTGCCTGGTATGACAATGAGTGGGGATACTCGCAGAGGGTGGT

TGATCTTGCTGATATTGTTGCAAGCAAGTGGAAATGA

CYP

>KAH6814225.1 rotamase CYP 7 [Perilla frutescens var. frutescens]

Length = 204 Score = 315 bits (808), Expect = 1e-086 Identities = 158/197 (80%), Positives = 171/197 (86%), Gaps = 12/197 (6%)

MAAIGRPLLLFTLFLFLAVLVQAKKSKEDLKEVTHKVYFDVEIDG

KPAGRITMGLFGKTVPKTAENFRALCTGEKGVGKHGKPLHYKGS

TFHRIIPSFMIQGGDFTLGDGRGGESIYGEKFADENFKIKHTGPGFL

SMANAGRDTNGSQFFITTVTTSWLDGRHVVFGKVLSGMDVVYK

MEAEGTQSGTPKRKVVVADSGELPL

ATGGCAGCAATCGGGAGGCCTCTTCTCCTTTTCACTCTCTTTCT

CTTCCTCGCCGTACTAGTTCAGGCGAAGAAATCAAAGGAAGAT

TTGAAGGAAGTGACTCATAAGGTCTATTTCGATGTCGAGATCG

ATGGCAAACCTGCTGGGCGTATTACCATGGGTCTATTTGGGAA

AACTGTTCCCAAGACAGCAGAAAATTTTAGAGCCTTGTGTACA

GGGGAGAAGGGTGTTGGCAAACATGGAAGCCTCTTCATTAC

AAGGGAAGCACTTTCCATAGAATTATACCTAGCTTTATGATTC

AAGGAGGAGACTTTACTCTTGGCGATGGAAGAGGCGGGGAAT

CAATTTACGGAGAGAAGTTTGCTGATGAGAATTTCAAGATCAA

GCATACTGGACCTGGGTTTCTGTCTATGGCAAATGCTGGGCGT

GACACCAATGGTTCTCAGTTTTTCATCACAACTGTGACAACCA

GCTGGTTGGATGGCCGTCATGTTGTCTTTGGAAAGGTACTATCT

GGAATGGACGTGGTGTATAAGATGGAAGCTGAAGGTACGCAG

AGTGGAACACCAAAGCGCAAAGTAGTGGTTGCAGACAGTGGC

GAACTCCCTTTGTAA

TUA

>KAH6803578.1 tubulin alpha-5 [Perilla frutescens var. frutescens]

Length = 449 Score = 852 bits (2200), Expect = 0.0 Identities =

412/450 (91%), Positives = 434/450 (96%), Gaps = 1/450 (0%)

MREIISIHIGQAGIQVGNSCWELYCLEHGIQPDGMMPSDTSVGVA

NDAFNTFFSETGSGKHVPRAIFVDLEPTVIDEVRTGSYRQLFHPEQ

LISGKEDAANNFARGHYTVGKEIVDLCLDRVRKLADNCTGLQGF

LVFNAVGGGTGSGLGSLLLERLSVDYGKKSKLGFTIYPSPQVSTA

VVEPYNSVLSTHSLLEHTDVAVLLDNEAIYDICRRSLDIERPTYTN

LNRLISQIISSLTTSLRFDGAINVDITEFQTNLVPYPRIHFMLSSYAP

VISAEKAYHEQLSVPEITNAVFEPSSMMAKCDPRHGKYMACCLM

YRGDVVPKDVNAAVATIKTKRTVQFVDWCPTGFKCGINYQPPTV

VPGGDLAKVQRAVCMISNNTAVAEVFSRIDHKFDLMYAKRAFV

HWYVGEGMEEGEFSEAREDLAALEKDYEEVGAEGVDDEEEGED

F

ATGAGAGAGATCATAAGCATACACATCGGGCAAGCTGGGATT

CAGGTGGGGAATTCATGCTGGGAGCTCTATTGCCTCGAGCATG

GCATTCAGCCCGATGGAATGATGCCTAGTGACACATCGGTGGG

TGTCGCAAATGATGCTTTCAACACCTTCTTTAGCGAGACTGGA

TCAGGAAAGCATGTCCCCAGAGCTATATTTGTTGATCTCGAAC

CAACCGTTATTGATGAAGTTAGGACAGGGAGTTATCGCCAGCT

CTTCCATCCTGAACAGCTTATCTCTGGAAAGGAAGATGCTGCC

AACAATTTCGCCCGAGGGCATTACACCGTGGGGAAAGAGATT

GTCGATTTATGTCTTGACAGAGTGAGGAAGTTGGCTGATAACT

GCACTGGTCTGCAAGGGTTCTTGGTTTTCAATGCTGTAGGTGGT

GGTACTGGTTCTGGATTGGGTTCGTTGCTTTTGGAGCGTTTATC

TGTGGACTATGGGAAGAAGTCGAAGCTTGGCTTCACCATCTAT
CCTTCACCCCAGGTGTCGACTGCGGTTGTTGAACCTTACAACA
GTGTTCTCTCCACTCATTCTCTTCTTGAACACACGGATGTTGCC
GTGCTGTTGGATAATGAAGCCATTTACGATATTTGCCGGAGGT
CATTAGATATTGAGAGGCCTACGTACACCAATTTGAATCGTTT
GATTTCCCAGATCATATCGTCCCTGACTACTTCTCTACGGTTTG
ATGGAGCCATCAATGTAGATATCACAGAGTTCCAGACCAACCT
TGTACCATATCCTCGTATCCATTTCATGTTGTCATCATATGCAC
CAGTGATCTCAGCTGAGAAAGCTTACCACGAGCAACTGTCAGT
CCCCGAGATCACAAATGCCGTGTTTGAGCCCTCAAGCATGATG
GCAAAATGTGATCCTCGTCATGGAAAATACATGGCCTGTTGCT
TGATGTACCGTGGAGACGTTGTTCCCAAGGATGTCAATGCAGC
TGTCGCCACAATCAAAACAAAACGGACTGTTCAGTTTGTCGAT
TGGTGCCCGACTGGCTTCAAGTGCGGAATCAACTACCAACCTC
CTACCGTGGTACCTGGGGGCGATCTTGCCAAAGTGCAGCGCGC
TGTGTGCATGATCAGCAACAACACGGCGGTTGCTGAAGTGTTC
TCACGCATCGACCACAAATTCGATCTCATGTATGCCAAGAGAG
CGTTTGTCCACTGGTACGTGGGAGAAGGAATGGAGGAAGGCG
AGTTCAGCGAAGCTCGTGAGGATTTGGCTGCGCTCGAGAAAGA
CTACGAAGAAGTCGGCGCAGAGGGTGTTGACGACGAGGAAGA
AGGTGAAGACTTCTAA

TUB

>KAH6773463.1 tubulin beta 8 [Perilla frutescens var. frutescens]

Length = 447 Score = 868 bits (2242), Expect = 0.0 Identities = 416/442 (94%), Positives = 436/442 (98%), Gaps = 1/442 (0%)

MREILHIQGGQCGNQIGAKFWEVICEEHGIDHTGRYNGESELQLE
RVNVYYNEASGGRYVPRAVLMDLEPGTMDSVRSGPYGQIFRPDN
FVFGQSGAGNNWAKGHYTEGAELIDSVLDVVRKEAENCDCLQG
FQVCHSLGGGTGSGMGTLLISKIREEYPDRMMLTFSVFPSPKVSD
TVVEPYNATLSVHQLVENADECMVLDNEALYDICFRTLKLATPTF
GDLNHLISATMSGVTCCLRFPGQLNSDLRKLAVNLIPFPRLHFFM
VGFAPLTSRGSQQYRALTVPELTQQMWDAKNMMCAADPRHGR
YLTASAMFRGKMSTKEVDEQMLNVQNKNSSYFVEWIPNNVKSS
VCDIPPRGLKMASTFIGNSTSIQEMFRRVSEQFTAMFRRKAFLHW
YTGEGMDEMEFTEAESNMNDLVAEYQQYQDATADDDEEYEEEN
EHEDVNA

ATGAGAGAAATATTGCACATCCAAGGCGGGCAATGCGGCAAC
CAAATCGGCGCCAAATTCTGGGAGGTGATTTGCGAGGAACAC
GGCATCGATCACACCGGGAGGTACAACGGCGAATCGGAGCTC
CAGCTGGAGCGCGTGAACGTCTACTACAATGAGGCCAGCGGC
GGGAGGTACGTCCCACGCGCCGTCCTCATGGACCTCGAGCCCG
GCACCATGGATTCCGTCAGATCCGGGCCCTACGGCCAGATCTT
CAGGCCCGATAACTTCGTCTTCGGGCAGTCCGGCGCCGGAAAT
AATTGGGCGAAGGGACATTACACCGAGGGAGCCGAGCTGATC

GACTCCGTGCTCGATGTCGTTCGCAAGGAGGCCGAGAATTGTG

ACTGCTTGCAAGGATTTCAAGTGTGTCATTCTTTGGGTGGAGG

CACTGGTTCTGGTATGGGCACCCTTCTCATCTCGAAAATCAGG

GAGGAGTACCCGGACAGAATGATGTTGACATTCTCCGTCTTTC

CATCTCCAAAGGTATCCGACACTGTTGTTGAGCCATACAACGC

CACGTTGTCAGTTCATCAGCTCGTAGAGAATGCAGACGAGTGT

ATGGTTTTGGATAACGAGGCTCTCTATGATATTTGCTTCCGTAC

TCTCAAACTTGCCACTCCTACATTTGGTGATCTCAACCACCTCA

TCTCCGCCACCATGAGTGGTGTTACATGTTGTCTCCGGTTCCCA

GGGCAGCTCAACTCGGATCTCAGGAAACTCGCTGTCAATCTTA

TCCCATTCCCACGACTTCATTTCTTTATGGTCGGATTTGCACCC

TTAACCTCTCGAGGCTCCCAGCAGTACAGGGCTCTCACAGTGC

CCGAACTCACTCAGCAGATGTGGGATGCGAAGAACATGATGT

GTGCTGCAGACCCGCGGCATGGACGCTACTTGACTGCCTCGGC

CATGTTCCGTGGCAAGATGAGCACCAAAGAAGTTGACGAGCA

GATGCTCAACGTTCAGAACAAGAATTCATCCTATTTTGTTGAGT

GGATTCCTAACAACGTGAAATCTAGTGTTTGTGACATTCCTCCC

AGGGGTCTGAAAATGGCTTCTACCTTCATTGGCAACTCCACCT

CGATCCAGGAGATGTTCAGGCGCGTTAGCGAGCAATTCACTGC

TATGTTCAGGCGCAAGGCTTTCTTGCACTGGTACACCGGTGAA

GGAATGGATGAAATGGAGTTCACCGAGGCTGAAAGCAACATG

AACGATCTTGTGGCGGAGTACCAGCAATACCAGGATGCGACG

GCCGATGATGATGAGGAGTATGAGGAAGAAAATGAACATGAA

GATGTTAATGCTTGA

PfLPCAT1.1:

>KAH6818197.1 MBOAT bound O-acyl family protein [Perilla frutescens var. frutescens] Length = 460

Score = 743 bits (1918), Expect = 0.0

Identities = 358/462 (77%), Positives = 403/462 (87%), Gaps = 4/462 (0%)

MDSMASAIGVSVPVLRFLLCFVVTIPVSFLHRFVPGGPTARHFYAA
ATGAILSYLSFGFSSNLHFIVPMLSGYASMVLCRKYCGIITFFLAFG
YLIGCHVYYMSGDAWKEGGIDATGALMVITLKIISCVINYNDGML
KEEDLREAQRKNRLLKLPSLVEYFGYCLCSGSHFAGPVYEMKDY
LEWTERKGIWQPSDKGLSPSPYFATFRALLQSAICMGLYLYLVPHF
PLSRFTEPLYQEYGFWKRLSYQYMSGFTARWKYYFIWSISEASTIIS
GLGFSGWTDSNPPKPKWDRAKNVDILGVELAKSAVQIPLVWNIQ
VSTWLRHYVVYERLIQKGKKPGFFQLLATQTVSAVWHGLYPGYIIF
FVQSALMIAGSRVLYRWQQASQADLLKKLLSVFNFSYTLLVLNYS
AVGFMVLSLHETLTAYGSVFYIGTVLPIVLIMLGKIIKPARPARSKA
RKEE

>lcl|QFCC02000001.1_cds_KAH6818197.1_1378 [locus_tag=C2S51_001800] [protein=MBOAT bound O-acyl family protein] [protein_id=KAH6818197.1] [location=complement(join(43224061..43224201,43224291..43224403,4

3224493..43224556,43225495..43225580,43226540..43226948,4322704 1..43227261,43227756..43227811,43228779..43229071))] [gbkey=CDS]

ATGGATTCCATGGCGTCGGCGATCGGAGTCTCGGTCCCCGTTCT
CCGCTTCCTGCTCTGCTTCGTCGTCACAATTCCAGTCAGCTTCC
TCCACCGATTCGTTCCCGGCGGTCCGACCGCCCGCCATTTCTAC
GCCGCCGCCACCGGGGCTATTCTGTCCTACCTCTCCTTCGGATT
CTCTTCAAATCTGCATTTCATCGTGCCGATGCTCTCAGGTTACGC
GTCGATGGTGCTTTGCCGAAAGTACTGTGGGATTATTACTTTTT
TCTGGCGTTTGGATACCTCATCGGATGCCATGTATACTACATGAG
TGGTGATGCGTGGAAGGAAGGAGGTATCGATGCTACAGGTGCT
CTGATGGTGATAACACTGAAAATAATATCATGTGTCATAAACTAC
AATGATGGAATGCTAAAAGAGGAGGATCTGCGTGAAGCACAAA
GGAAAAACCGGTTGCTCAAGCTGCCATCATTAGTCGAGTACTTT
GGTTACTGTCTCTGCAGTGGAAGTCACTTTGCTGGTCCTGTGTA
TGAAATGAAAGATTATCTTGAATGGACAGAGAGGAAAGGGATT
TGGCAACCTTCGGACAAGGGATTGTCTCCCTCTCCCTATTTTGC
AACTTTCAGGGCTCTTCTACAATCTGCTATCTGCATGGGCTTGTA
TCTGTATCTTGTACCACATTTCCCACTCTCTCGATTCACAGAACC
ATTGTACCAAGAATATGGGTTCTGGAAGCGGTTAAGTTACCAGT
ACATGTCAGGATTCACTGCTCGTTGGAAATATTATTTTATCTGGT
CCATTTCAGAAGCTTCTACTATTATTTCCGGCCTGGGATTCAGTG
GCTGGACAGATTCAAATCCACCCAAACCCAAATGGGACCGTGC

AAAAAATGTTGACATATTAGGTGTTGAGTTGGCTAAGAGTGCAG

TGCAGATACCACTTGTGTGGAATATTCAAGTCAGCACCTGGCTT

AGGCACTATGTTTATGAGAGGCTGATTCAGAAAGGTAAGAAAC

CGGGATTCTTTCAGTTGCTGGCTACTCAAACTGTGAGTGCAGTA

TGGCATGGATTGTATCCGGGCTATATCATATTCTTTGTTCAGTCA

GCTTTGATGATTGCTGGTTCAAGAGTTCTCTACAGATGGCAGCA

AGCCAGCCAAGCAGATCTACTTAAGAAGCTGCTCAGTGTGTTC

AACTTCAGCTACACGCTTCTGGTGCTGAACTATTCAGCCGTTGG

TTTCATGGTGCTAAGTCTGCACGAGACGCTAACTGCATATGGGA

GTGTCTTCTACATTGGCACTGTTCTTCCTATTGTCTTGATCATGC

TAGGCAAAATCATCAAGCCCGCGAGGCCAGCCCGATCGAAAGC

CCGAAAAGAGGAATGA

PfLPCAT1.2:

>KAH6790771.1 MBOAT bound O-acyl family protein [Perilla

frutescens var. frutescens] Length = 465

Score = 741 bits (1914), Expect = 0.0

Identities = 351/463 (75%), Positives = 405/463 (87%), Gaps = 4/463 (0%)

MELPEMDSMASAIGVSVPVLRFLLCFAATIPVSFFHRFVPGGPAAR

HFYAAATGAFLSYLSFGLSSNLHFIVPMLLGYASMLLCRNYCGILT

FFLAFGYLIGCHVYYMSGDAWKEGGIDATGSLMVITLKIISCVINY

NDGLLKEEDLREAQKRNRLLKLPSLLEYFGYCLCCGSHFAGPVYE

MKDYLEWTERKGIWQPTKQGPSPSPYLPTFRALLQASVCMGLYL

YLVPHFPLSRFTEPVYQEYGFWKRLGYQYMSGFTARWKYYFIWSI
SEASVIISGLGFCGWTESSSPKPRWDRAKNVDILGVELAKSSVQLP
LVWNIQVSTWLRHYVYERLIQNGKKPGFFQLLATQTVSAVWHGL
YPGYIFFFVQSATMIAGSRVLYRWQQASQSALVKNLLVLVNFAYTL
LVLNYSCVGFMVLSLHETLAAYGSVYYIGTILPIVLILLGKVFKPG
RPARSKARKDQ

>lcl|QFCC02000012.1_cds_KAH6790771.1_8158
[locus_tag=C2S51_005777] [protein=MBOAT bound O-acyl family protein] [protein_id=KAH6790771.1] [location=join(66141068..66141375,66142956..66143011,66143739..66143959,66144051..66144459,66146035..66146120,66146774..66146837,66146931..66147043,66147131..66147271)] [gbkey=CDS]
ATGGAGCTGCCGGAGATGGATTCCATGGCCTCCGCCATCGGAGT
ATCGGTGCCGGTGCTCCGCTTCCTACTCTGCTTCGCCGCAACCA
TCCCCGTCAGCTTCTTCCACCGCTTCGTTCCAGGGGGGCCGGC
GGCGCGCCACTTCTACGCCGCCGCTACAGGAGCTTTCCTCTCGT
ACCTTTCCTTTGGATTGTCGTCGAATTTGCATTTCATTGTGCCGA
TGCTTTTAGGATACGCGTCGATGCTGCTTTGCCGGAACTACTGT
GGGATCCTTACTTTCTTCCTCGCGTTTGGTTACCTCATTGGATGC
CATGTGTATTACATGAGTGGTGATGCATGGAAGGAAGGGGGTAT
AGATGCTACAGGTTCTCTGATGGTGATAACACTAAAAATCATATC
ATGTGTCATTAATTACAATGATGGATTGCTGAAAGAGGAAGATT

TACGTGAGGCTCAAAAGCGAAACCGGTTGCTAAAGCTGCCATC

TTTACTAGAGTACTTTGGTTACTGTCTCTGTTGTGGAAGTCACTT

TGCTGGTCCAGTGTATGAAATGAAAGATTATTTGGAATGGACTG

AGAGAAAAGGGATTTGGCAACCTACAAAGCAGGGACCATCTCC

CTCTCCTTATTTGCCAACTTTCAGGGCTCTTTTGCAAGCTTCTGT

CTGTATGGGATTATATCTATATCTCGTACCACATTTCCCACTTTCA

CGATTCACAGAACCAGTATACCAAGAATATGGCTTCTGGAAACG

ATTAGGCTACCAATACATGTCAGGCTTCACTGCTCGTTGGAAAT

ATTATTTCATCTGGTCTATCTCAGAAGCTTCCGTTATTATTTCTGG

CCTTGGATTTTGTGGCTGGACTGAATCAAGTTCACCCAAACCGC

GATGGGACCGTGCAAAGAATGTTGATATATTGGGGGTTGAGTTG

GCAAAGAGCTCCGTGCAGTTACCACTTGTTTGGAATATTCAAGT

CAGCACCTGGCTTAGGCACTATGTCTATGAGAGGTTGATTCAGA

ATGGAAAGAAACCGGGCTTCTTCCAGTTGCTGGCCACCCAAAC

TGTCAGTGCAGTCTGGCATGGATTATATCCCGGGTATATCTTCTT

TTTCGTTCAATCTGCTACGATGATTGCTGGCTCAAGAGTTCTTTA

CAGATGGCAGCAAGCTAGCCAATCAGCTCTGGTTAAAAATCTG

CTTGTGCTAGTAAACTTTGCTTACACACTTCTTGTTCTGAACTAC

TCCTGCGTTGGTTTCATGGTACTAAGCCTGCACGAAACACTAGC

TGCCTACGGAAGTGTTTACTACATCGGAACCATCCTTCCAATAG

TTCTAATCCTGCTTGGCAAAGTCTTTAAGCCGGGAAGGCCAGC

ACGATCAAAAGCTCGAAAAGACCAGTAG

PDAT:

>Locus_7255_Transcript_6/8_Confidence_0.545_Length_2638

117 9e-025

TGCAATCCCTCAAAAAAGCTCGAAATAGTTTCAAAGAAAAGC

ATACATACGTACAGCACGAGCAGCAACTAGGTACTAATCATTT

TATCGATCATCTCTGGCTCTCTGTCATAACCGATTCAATTCATT

CGCGAAAACTAATTCCGATCGTTGGATTTCCCTGCGGTAGCTG

TTCGCGATGCCATCGTTGCGAAGGAGACGCGCCGCCGGAGGG

GAGGCAGTCCGAGCACCGGATCCGGATGAGAAGAAGCCGGAG

CTCCGGAAACCGAGGAAGCAGCAAAAATGGTCGTGCGTGGAT

AGTTGCTGCTGGCTTATTGGGTGCATATGTGTAGTGTGGTGGG

GGCTGTTGTTTTCCTACAATATGATGCCTTCTTCATTCCCGCAA

TACGTAACGGAGGCGATAACCGGACCAATGCCCGACCCGCCG

TGGGTGAAGCTGAAGAAGGAAGGGCTGGAGGCGAAGCATCCG

GTGGTGTTCGTGCCCGGGATTGTGACGGGAGGGCTGGAGCTGT

GGGAGGGGCACAGTTGTGCTGAAGGATTGTTCAGAAAGAGGC

TGTGGGGAGGCACATTCGGCGAAGTTTACAAGAGGCCACTATG

CTGGGTGGAGCATATGTCATTGGATAATGAAACTGGCTTGGAT

CCTCCTGGTGTAAGAGTCAGGCCTGTTACTGGTCTTGTTGCTGC

AGATTACTTTGCTCCAGGATATTTTGTTTGGGCAGTTCTCATAG

CTAGTCTTGCTCGGATTGGGTATGAAGAGAAAGCCATGTACAT

GGCTGCATATGATTGGAGGCTTTCATTCCAGAACACAGAAGTG

CGTGATGGAACACTGAGTAGAATAAAGAGTAATATTGAACTG
ATGGTTGCTACAAGTGGACACAGGGCTGTAATCATTCCACATT
CCATGGGTGTTCTATACTTTCTGCATTTTATGAAGTGGGTGGAG
GCACCTGCTCCAATGGGGGGAGGAGGCGGACCGGATTGGTGT
GCTAAACACATAAAGGCTGTGATGAACATTGGAGGGCCGCTTT
TAGGTGTTCCAAAAGCTGTTTCTGGGCTTTTCTCTGCAGAAGCA
AGAGATATTGCTGTTGCCAGGGCCATTGCACCAGGTGTCCTGG
ATAAGGATCTATTTCGAATCCAAACCTTGCAGCACATAATGAA
GATGACCAGAACATGGGATTCAACAATGTCAATGATACCAAA
AGGTGGAGAAACCATATGGGGTGGACTTGACTGGTCACCTGA
ACAGGGTTATGCTGTAAGCAAAAGAAAACACAATACCAACCA
ATCCGAGAATGCAGCCAGTACTGAAGATGGGAATGTTGAACC
AAATGCAAAACATGCTCATTATGGAAGAATGATATCGTTTGGG
AAAGATGTAGCTGAGGCAGATTCTTCTAAGATTGAGAGGATTG
ATTTTAGGGATGCTGTAAAGGGTAACAATTTTGGAAACAATAC
TTGTGGAGATGTGTGGACAGAGTATCATGACATGGGTATTAGT
GGCATCACGGCCATTGCTGAATATAAAGCTTATACTGCTGGAG
ATATACTGGATCTGCTAAACTATGTTGCTCCCAAAATGATGGA
GCGTGGCAGTGCTCATTTTTCTTATGGGATTGCTGATAACTTGG
ATGATCCCAAGTATACACACTACAAATATTGGTCAAATCCATT
GGAAACAAGGTTACCAAATGCTCCCGACATGGAGGTCTTCTCT
ATGTATGGTACTGGCATTCCAACTGAAAGAGCTTATGTATACA

AGCAGGTTCCGGCTGCAGAGTGCTACATTCCATTTCAAATTGA

TACATCTGCAGTTGAAGAAGATAAAGATAGATGCATAAAAGA

TGGTGTTTTTACCGTTGACGGTGATGAAACTGTGCCTATTCTAA

GTGCAGGCTTCATGAGTGCTAAAGGGTGGCGTGGAAAAACTA

GATTTAATCCTTCGGGAATGAAGAATTATGTAAGGGAGTACGA

CCATGCTCCTCCAGCCAATCTTCTTGAAGGCCGTGGTACCCAG

AGTGGTGCTCATGTTGATATCATGGGAAATTTTGCTTTGATAGA

GGACGTTATGAGGGTTGCTGCCGGTGCTACAGGGGACGAGCTG

GGAGGTGATCGTGTGTTCTCAGACATCTTCAAGTGGTCCGACA

AGATCGATCTACCTCTATAAAGGTAACCTGTACGATAGACTTT

CAAGGCTTCATCCCATAAATTACACAAACGGTCATCTTGAGGT

AGAATGGTGCACCTCAGTTGAGGATTCCATTCTGTTACGTCAA

ATTTTAGTCGGTCAGCTAATTGTTCCGTAGGCTCGACTATGCAG

TGACATGGTATGATGCATGAGGCGAAGTACAGATATAGACGTT

TGCTTCAGCAAGATCGAGCTTTTAAAAGTAGCATACCGCATAT

TTGGTGGTTAGACTATTATATCTTATACCAGGATTGGTGAAAA

CGAGGGCGATTGACCTCCATACATATCTCTACATTATGTCGCT

GATTATCTGATTTATTTTTCCCTCTCAAACTGATATTTTGTATTA

TATCATTGCACTTTTGCTCTGTTGGATCAAGAAACAGGGGAGA

GAGAGTGAGGAGGTGTATTTGTTAATTGGGTTAAATTAGAATT

CTTCATTTTTATCTGGAC

> CDS

ATGCCATCGTTGCGAAGGAGACGCGCCGCCGGAGGGGAGGCA

GTCCGAGC

ACCGGATCCGGATGAGAAGAAGCCGGAGCTCCGGAAACCGAG

GAAGCAGC

AAAAATGGTCGTGCGTGGATAGTTGCTGCTGGCTTATTGGGTG

CATATGT

GTAGTGTGGTGGGGGCTGTTGTTTTCCTACAATATGATGCCTTC

TTCATT

CCCGCAATACGTAACGGAGGCGATAACCGGACCAATGCCCGA

CCCGCCGT

GGGTGAAGCTGAAGAAGGAAGGGCTGGAGGCGAAGCATCCGG

TGGTGTTC

GTGCCCGGGATTGTGACGGGAGGGCTGGAGCTGTGGGAGGGG

CACAGTTG

TGCTGAAGGATTGTTCAGAAAGAGGCTGTGGGGAGGCACATTC

GGCGAAG

TTTACAAGAGGCCACTATGCTGGGTGGAGCATATGTCATTGGA

TAATGAA

ACTGGCTTGGATCCTCCTGGTGTAAGAGTCAGGCCTGTTACTG

GTCTTGT

TGCTGCAGATTACTTTGCTCCAGGATATTTTGTTTGGGCAGTTC

TCATAG

CTAGTCTTGCTCGGATTGGGTATGAAGAGAAAGCCATGTACAT

GGCTGCA

TATGATTGGAGGCTTTCATTCCAGAACACAGAAGTGCGTGATG

GAACACT

GAGTAGAATAAAGAGTAATATTGAACTGATGGTTGCTACAAGT

GGACACA

GGGCTGTAATCATTCCACATTCCATGGGTGTTCTATACTTTCTG

CATTTT

ATGAAGTGGGTGGAGGCACCTGCTCCAATGGGGGGAGGAGGC

GGACCGGA

TTGGTGTGCTAAACACATAAAGGCTGTGATGAACATTGGAGGG

CCGCTTT

TAGGTGTTCCAAAAGCTGTTTCTGGGCTTTTCTCTGCAGAAGCA

AGAGAT

ATTGCTGTTGCCAGGGCCATTGCACCAGGTGTCCTGGATAAGG

ATCTATT

TCGAATCCAAACCTTGCAGCACATAATGAAGATGACCAGAAC

ATGGGATT

CAACAATGTCAATGATACCAAAAGGTGGAGAAACCATATGGG

GTGGACTT

GACTGGTCACCTGAACAGGGTTATGCTGTAAGCAAAAGAAAA

CACAATAC

CAACCAATCCGAGAATGCAGCCAGTACTGAAGATGGGAATGT

TGAACCAA

ATGCAAAACATGCTCATTATGGAAGAATGATATCGTTTGGGAA

AGATGTA

GCTGAGGCAGATTCTTCTAAGATTGAGAGGATTGATTTTAGGG

ATGCTGT

AAAGGGTAACAATTTTGGAAACAATACTTGTGGAGATGTGTGG

ACAGAGT

ATCATGACATGGGTATTAGTGGCATCACGGCCATTGCTGAATA

TAAAGCT

TATACTGCTGGAGATATACTGGATCTGCTAAACTATGTTGCTCC

CAAAAT

GATGGAGCGTGGCAGTGCTCATTTTTCTTATGGGATTGCTGATA

ACTTGG

ATGATCCCAAGTATACACACTACAAATATTGGTCAAATCCATT

GGAAACA

AGGTTACCAAATGCTCCCGACATGGAGGTCTTCTCTATGTATG

GTACTGG

CATTCCAACTGAAAGAGCTTATGTATACAAGCAGGTTCCGGCT

GCAGAGT

GCTACATTCCATTTCAAATTGATACATCTGCAGTTGAAGAAGA

TAAAGAT

AGATGCATAAAAGATGGTGTTTTTACCGTTGACGGTGATGAAA

CTGTGCC

TATTCTAAGTGCAGGCTTCATGAGTGCTAAAGGGTGGCGTGGA

AAAACTA

GATTTAATCCTTCGGGAATGAAGAATTATGTAAGGGAGTACGA

CCATGCT

CCTCCAGCCAATCTTCTTGAAGGCCGTGGTACCCAGAGTGGTG

CTCATGT

TGATATCATGGGAAATTTTGCTTTGATAGAGGACGTTATGAGG

GTTGCTG

CCGGTGCTACAGGGGACGAGCTGGGAGGTGATCGTGTGTTCTC

AGACATC

TTCAAGTGGTCCGACAAGATCGATCTACCTCTATAA

DGAT:

>Locus_14696_Transcript_3/7_Confidence_0.583_Length_1981

96 3e-018

GCCCCATATAGTGACTTAGAATTTACAAATTGTCATACAGCCC

AGAATGTAAATATACAAATTCAACCCCATGAGCACTCACCACA

GCGTTAAAATGTCAGTAAACGAGGGGTATGCTTCATCAAATTT

TCACCATTTCTGTATTCCTTGAAAAATAGACAAAATTTGCATGC

CCCCTTACCCCCATCACAACAGATCATAACGTCGTCCTAGGTG

AAGGCAGATAACAAACTACTCCCAGCTATTTAAGAAACATAGT

TGTAGCAAATTATCTACCAGCTCACAATCAGGATCCTTGCTCCT

TTTTGTTTTTCGGGTGGGTTGGGGCTGGTGAGCTTTAGATTTCT

ACTCAGAAGATGAATCACATCCCTACCTTGCACTAGCTTTTCG

ATTCATCAAGTCGTGGTAGTACAGCAACACACACATAGGTTGA

CCAAAGATACAGAAGAAGCACCAGAACATCATATTGCCCACC

ATTGAGTTTTTGAACTTTTCTTGCAAGTAATTAGTCACGATTAC

GAGAGGAACCTGAAGCATGATACCCGAGAACGCCCAAAACTT

GAATATTTGGCAAGGGACTGCAACGCACAGCTCATGAAAAAT

CGCAGACACAAGAAACGCGATCAAAACTGCCACTATCTTTGGT

ATTCCATTTTGTAAGCATGGACAGTATATATGCCGAACCATCC

ATTTATGGACAGGCATATTCCACATTCTCCAGTACTCCTCCACT

GTCCTCGCATTCCACCAATCCTTATAAAACTCACGGTCCCCAA

AGCACAGAAGTTCAGCAAGTATATTTAGCCAGAGGTGGAAAA

AACAATAAAACATGCAGAGCCACACATATAAATTTGGAACAG

AAAGCTTCAAGACCCTCTCAATGGCATATAAAAGGTTTCCTTT

CAGAGGATGTTGAGAATTTTGAACGATCGGGTTTATGTACTGT

TCTATAATAAATCCCATGAGTCCTGTGAATATTACCAGCTTAAT

TAGTTGCCTTACCACCCAACCCTTCCGAATGCAAGCTGTCCGA

GGGTAGCTTGGCTGGTAACACAATGTAGGAGCAACCATGAAG

TATGCCAGACTCTTAAAGCTTACATCATAAGCGTAGTCGAGGT

TCCAGTACCTGGACATAGCTTCCCACTTATCAAGTGATTTTGCA
AGTACTCTCAAATCATAGTTTGTATGTGCGTAGGAAACGAGCT
TCAACCATACAGTGCAAGCAAAGAGCATTAGCGTGACACCTG
ATAGAACAGCAGAATCACACCTAAGAATGACAACAACTGGAA
ACAAGATTTCCACTGTTGTGATTGTAACATGAAGAAAGACTGC
GACCCACTCAGGTATATAATTTAGTTTCACCAACTTCTCGACA
AGAAATGAAGCGAGTGCAAAAACTGGAAGACTAAGACAACAC
ATTAGCAGTGGCCAATCCCTAAGCGATGTTGAACTAAACCAAA
ATCCTGATTTGATCAGCCACCCATACTTCATTAAATTCTCAATT
ATTAGTCTGCTATTTACAGCAACAAGCACCACTATACAAAGGT
TGAAGAGGCCTGCATGGCTCTGTTTGAAGATGGCGTCGGAGCT
AAGAGGACTCTCCTTATTTTTGCGGTGAGCAGGCGCCGCCGGC
CTGAATGTGAATTTGACGGCCATAACATCAGTTCCGTTGCCAT
TACTAGTTTCTCCATTCTCCTTCACTTTCGCCCCCTCCTCCTTAC
CATAACTCTCCTGTTTTTCGTTCTCGGATTCCACGGCTCCGCCG
CGGAGATTTTCGATCAAATTAGCGTCGTTTCGAACATTTTCGGA
ATCATTGATTGCGCTCTCTGCCTCCAGAGAGTTGGAATCGGAG
TCGAGAAGAGGCGGAACGGAGCGCGCACTTTGTCTCCGGCGA
AGAGTGGTGTGATGTGCGGCGCCGTTGTCGGCACTGGACGACG
TCGTGTCCAGGATCTCCGGCGAGTCCAAGATCGCCATCGCACG
G

> CDS

ATGGCGATCTTGGACTCGCCGGAGATCCTGGACACGACGTCGT

CCAGTGC

CGACAACGGCGCCGCACATCACACCACTCTTCGCCGGAGACAA

AGTGCGC

GCTCCGTTCCGCCTCTTCTCGACTCCGATTCCAACTCTCTGGAG

GCAGAG

AGCGCAATCAATGATTCCGAAAATGTTCGAAACGACGCTAATT

TGATCGA

AAATCTCCGCGGCGGAGCCGTGGAATCCGAGAACGAAAAACA

GGAGAGTT

ATGGTAAGGAGGAGGGGGCGAAAGTGAAGGAGAATGGAGAA

ACTAGTAAT

GGCAACGGAACTGATGTTATGGCCGTCAAATTCACATTCAGGC

CGGCGGC

GCCTGCTCACCGCAAAAATAAGGAGAGTCCTCTTAGCTCCGAC

GCCATCT

TCAAACAGAGCCATGCAGGCCTCTTCAACCTTTGTATAGTGGT

GCTTGTT

GCTGTAAATAGCAGACTAATAATTGAGAATTTAATGAAGTATG

GGTGGCT

GATCAAATCAGGATTTTGGTTTAGTTCAACATCGCTTAGGGATT

GGCCAC

TGCTAATGTGTTGTCTTAGTCTTCCAGTTTTTGCACTCGCTTCAT

TTCTT

GTCGAGAAGTTGGTGAAACTAAATTATATACCTGAGTGGGTCG

CAGTCTT

TCTTCATGTTACAATCACAACAGTGGAAATCTTGTTTCCAGTTG

TTGTCA

TTCTTAGGTGTGATTCTGCTGTTCTATCAGGTGTCACGCTAATG

CTCTTT

GCTTGCACTGTATGGTTGAAGCTCGTTTCCTACGCACATACAA

ACTATGA

TTTGAGAGTACTTGCAAAATCACTTGATAAGTGGGAAGCTATG

TCCAGGT

ACTGGAACCTCGACTACGCTTATGATGTAAGCTTTAAGAGTCT

GGCATAC

TTCATGGTTGCTCCTACATTGTGTTACCAGCCAAGCTACCCTCG

GACAGC

TTGCATTCGGAAGGGTTGGGTGGTAAGGCAACTAATTAAGCTG

GTAATAT

TCACAGGACTCATGGGATTTATTATAGAACAGTACATAAACCC

GATCGTT

CAAAATTCTCAACATCCTCTGAAAGGAAACCTTTTATATGCCA

TTGAGAG

GGTCTTGAAGCTTTCTGTTCCAAATTTATATGTGTGGCTCTGCA

TGTTTT

ATTGTTTTTCCACCTCTGGCTAAATATACTTGCTGAACTTCTG

TGCTTT

GGGGACCGTGAGTTTTATAAGGATTGGTGGAATGCGAGGACA

GTGGAGGA

GTACTGGAGAATGTGGAATATGCCTGTCCATAAATGGATGGTT

CGGCATA

TATACTGTCCATGCTTACAAAATGGAATACCAAAGATAGTGGC

AGTTTTG

ATCGCGTTTCTTGTGTCTGCGATTTTTCATGAGCTGTGCGTTGC

AGTCCC

TTGCCAAATATTCAAGTTTTGGGCGTTCTCGGGTATCATGCTTC

AGGTTC

CTCTCGTAATCGTGACTAATTACTTGCAAGAAAAGTTCAAAAA

CTCAATG

GTGGGCAATATGATGTTCTGGTGCTTCTTCTGTATCTTTGGTCA

ACCTAT

GTGTGTGTTGCTGTACTACCACGACTTGATGAATCGAAAAGCT

AGTGCAA

GGTAG

FAD2:

>Locus_733_Transcript_8/17_Confidence_0.239_Length_1501 117 6e-025

TTTTTTTGAAGAAAAGGCTTTGCTTTGACCTCAACAAAAAAA
AACATAAATAGATCTTACAAAGTCTTATTCATCCAAAATTAAC
CAAGACTTTTTAGAACCCAACACAGTTCTAACTAAGCCTTATT
CAACCCACATGAACCAAGACTTCTTACCTTAACTTAAACAAAG
ACACAACCTAGATTATCGTTAACCACAACGAAAGCATAATTTA
ACCACCTACAACAACAGCCCAGAAAACTGCATTTCCTGCATAT
TTATCGCATAATCTAAAGCTTGTTATTGTACCAGAACACACCTT
TGTTCTTGTCACCTTCATCGGGCTCGACGTAGACGCACTCCTTC
GCTTCTCTCCACACGGCCTTAGCCACCGGGGTCCCGTCGAACT
GGTAGTACTCGCCTAGGATTGGTTTGATCGCCTTCGTCGCCTCC
ATCGCGTGGTAGTGCGGCATGGTAGAGAACAAATGGTGCGCG
ACGTGGGTGTCTGTTATGTTATGGAAGACGGTGTTGAGAACGC
CGTAATCTCTGTCGACCGTGGACAGAGCGCCTCGCAGCCAGTC
CCACTCCGACGAGTCATAATGCGGGAGGGAGGCGTGGGTGTG
CTGTAAGTACGTGATCAAGACGAGGAACCCGTTCACCACGAGC
AACGGGACACCATACACACAGAGAACCCAGGCGAGCCCTTTA
GCAAGGGTCAAACGGTACAGCCCGTAGAGCATGGCGAGAATG
CCCACGTCCGAGAGGAAGATCTGAACGCGCTCGCGGTCAGAG

TAAATCGGGCTCTTAGGGTCGAAGTGGCATGCGAACCGGTCGT

AGGGCCTTCCGGAGACATTGAACATGAGATACAAAGGCCAGC

CGAGGGTGAACTGGACTATGAGAGTGAGGATTCTACCGGGTG

GGTTGTTTAGGTACTTGGCAGACGAGCCGAGTGCCGACTTGAC

CTTGGGTACAAACACCTCGTCCCGCTCGAGGGAACCGGTGTTG

GAGTGGTGGCGGCGGTGGCTGTACTTCCAAGAGAAGTAGGGG

ACGAGGAGAAACGAGTGGAGGACTAGGCCGACGGTGTCGTCC

AGCCATTGGTAGTCACTGAAGGCATGGTGGCCACATTCATGGG

CTATGACCCAAACACCAGTTAGAATGCAACCTTGGCATATCAT

ATAAAGAGGCCAGGCTACGTAGGAGAGAGGGTAAGGGAGTTG

ATGGAAATAGTTTGTGGCGACGTAGTAAAACAGAGAGGCAAT

GACGAGGTCGTATAGGACATAGGAAAAGGAACGAGGAATGGA

TCGCTGAACAATGGGGTGGAATGGCTTTCTTTATCTCACCGAG

CGTGAATGGAGGCTTTGTGAATGGAACTCGTTCGACAACAGAT

TTTGCCTTCTTACCCTCTCTCCTACGTGGCCTGGCCTCTTTATAT

GATATGCCAAGGTTGCATTCTAACTGGTGTTTGGGTCATAGCC

CATGAATGTGGCCACCATGCCTTCAGTGACTACCAATGGCTGG

ACG

> CDS

ATGATATGCCAAGGTTGCATTCTAACTGGTGTTTGGGTCATAG

CCCATGA

ATGTGGCCACCATGCCTTCAGTGACTACCAATGGCTGGACGAC

ACCGTCG

GCCTAGTCCTCCACTCGTTTCTCCTCGTCCCCTACTTCTCTTGG

AAGTAC

AGCCACCGCCGCCACCACTCCAACACCGGTTCCCTCGAGCGGG

ACGAGGT

GTTTGTACCCAAGGTCAAGTCGGCACTCGGCTCGTCTGCCAAG

TACCTAA

ACAACCCACCCGGTAGAATCCTCACTCTCATAGTCCAGTTCAC

CCTCGGC

TGGCCTTTGTATCTCATGTTCAATGTCTCCGGAAGGCCCTACGA

CCGGTT

CGCATGCCACTTCGACCCTAAGAGCCCGATTTACTCTGACCGC

GAGCGCG

TTCAGATCTTCCTCTCGGACGTGGGCATTCTCGCCATGCTCTAC

GGGCTG

TACCGTTTGACCCTTGCTAAAGGGCTCGCCTGGGTTCTCTGTGT

GTATGG

TGTCCCGTTGCTCGTGGTGAACGGGTTCCTCGTCTTGATCACGT

ACTTAC

AGCACACCCACGCCTCCCTCCCGCATTATGACTCGTCGGAGTG

GGACTGG

CTGCGAGGCGCTCTGTCCACGGTCGACAGAGATTACGGCGTTC

TCAACAC

CGTCTTCCATAACATAACAGACACCCACGTCGCGCACCATTTG

TTCTCTA

CCATGCCGCACTACCACGCGATGGAGGCGACGAAGGCGATCA

AACCAATC

CTAGGCGAGTACTACCAGTTCGACGGGACCCCGGTGGCTAAGG

CCGTGTG

GAGAGAAGCGAAGGAGTGCGTCTACGTCGAGCCCGATGAAGG

TGACAAGA

ACAAAGGTGTGTTCTGGTACAATAACAAGCTTTAG

FAD3:

>Locus_5107_Transcript_3/8_Confidence_0.600_Length_1089 60

1e-007

>Locus_22029_Transcript_15/22_Confidence_0.349_Length_1477

CCAAAATTAGCACACTCCCTCCTCTCTCCTCCACAAATCTCAGT

TACACACACATCGCCCCCCTCTCTCTCTAAACACTTTCCTCTCT

CTCTATATATATGCCAACCTCATGTATTCCATTTGTTCTAACTG

AAACTCGCTAGTTTATTGATTCAAACCCTCCCCAAAAGAATGG

CCGTTTCTTCCGGTGCCCGCCTCTCGAAGAGTGGCGCTGATGG

AGAGGTTTTCGACGGCCAACAACAATACGAAGGAATCGGAAA

GCGTGCGGCCGACAAATTCGACCCCGCCGCGCCGCCGCCCTTC

AAGATCGCCGACATCCGAGCGGCCATACCGGCGCATTGCTGGG

TGAAGAGTCCGTGGCGCTCATTGAGCTACGTCGTTTGGGACGT

CGCCGCCGTATTCGCGCTGCTCGCCGCCGCCGTTTACATCAAC

AGCTGGGCGTTTTGGCCGGTTTACTGGATTGCTCAGGGCACCA

TGTTTTGGGCCCTTTTCGTTCTTGGGCATGATTGTGGGCACGGG

AGTTTTTCAGACAATACCACGCTGAATAACGTGGTGGGACATG

TGCTTCACTCATCAATTCTTGTACCGTATCATGGATGGCGAATC

AGCCATAGAACTCACCACCAAAACCATGGACATGTCGAGAAG

GACGAGTCGTGGGTGCCGTTGCCTGAGAATTTGTACAAGAAGT

TGATTTTTCCACCAAATTCTTGAGATACAAAATCCCATTCCCCA

TGTTTGCATATCCTTTATATTTGTGGTATAGAAGTCCGGGAAAA

ACTGGATCTCACTTCAACCCTTACAGCGATTTGTTTAAACCAA

ATGAGAGGGGCTTGATAGTGACTTCAACAATGTGCTGGGCTGC

AATGGGTGTTTTCCTCCTCTATGCCTCCACCATTGTTGGTCCAA

ACATGATGTTCAAGCTCTACGGCGTACCATATTTGATATTCGTG

ATGTGGTTGGACACGGTAACATACTTACACCACCACGGTTATG

ACAAGAAACTCCCTTGGTACCGCAGCAAGGAATGGAGTTATTT

ACGAGGAGGATTGACGACCGTAGATCAAGATTATGGATTTTTT

AATAAAATTCACCACGATATTGGCACCCATGTTATACACCATC

TATTCCCTCAGATCCCACATTACCACTTAGTGGAGGCGACAAG

GGAGGCGAAAAGGGTGCTGGGGAATTACTACAGGGAGCCCAG

AAAATCTGGGCCAGTTCCACTGCACTTAATTCCTGCCTTGTTGA

AAAGTCTTGGTAGAGATCATTATGTTAGTGATAATGGAGACAT

AGTTTATTATCAAACAGATGATGAGCTCTTTCCTTCCAAAAAG

ATTTAGTGATGGACTCTTGATTGCCAAATTAGATTTAATTTACA

GTAGTCCTTTGTGCCACAATATTTTTGGAAGGAAAGAGCTCAT

CATCTGTTTGATAATAAACTATGTCTCCATTATCACTAACATAA

TGATCT

> CDS

ATGTTTGCATATCCTTTATATTTGTGGTATAGAAGTCCGGGAAA

AACTGG

ATCTCACTTCAACCCTTACAGCGATTTGTTTAAACCAAATGAG

AGGGGCT

TGATAGTGACTTCAACAATGTGCTGGGCTGCAATGGGTGTTTT

CCTCCTC

TATGCCTCCACCATTGTTGGTCCAAACATGATGTTCAAGCTCTA

CGGCGT

ACCATATTTGATATTCGTGATGTGGTTGGACACGGTAACATAC

TTACACC

ACCACGGTTATGACAAGAAACTCCCTTGGTACCGCAGCAAGGA

ATGGAGT

TATTTACGAGGAGGATTGACGACCGTAGATCAAGATTATGGAT

TTTTTAA

TAAAATTCACCACGATATTGGCACCCATGTTATACACCATCTA

TTCCCTC

AGATCCCACATTACCACTTAGTGGAGGCGACAAGGGAGGCGA

AAAGGGTG

CTGGGGAATTACTACAGGGAGCCCAGAAAATCTGGGCCAGTTC

CACTGCA

CTTAATTCCTGCCTTGTTGAAAAGTCTTGGTAGAGATCATTATG

TTAGTG

ATAATGGAGACATAGTTTATTATCAAACAGATGATGAGCTCTT

TCCTTCC

AAAAAGATTTAG
