# Supplementary material for: Selection and Validation of Reference Genes for RT-qPCR Analysis of Lipid Biosynthesis-Related Genes in Perilla frutescens
Source: Plants (Basel). 2026 Aug 25;15(17):2588. doi: 10.3390/plants15172588 (PMC13567130; doi:10.3390/plants15172588)
Supplement: Supplementary file 1 [file plants-15-02588-s001.zip › Table S1.pdf]

Table S1, primer sequences and amplicon information

Reference gene	Primer sequences (FP+RP)	Product Length (bp)
Actin	F:GCCGAAGCCGAGGATATTCA<br>R:ATGCCAACCATCACACCAGT	200
18S	F:TGCGTCTATGACTCGACTGC<br>R:ACGTTCTACCCTCGACAGGA	203
EF-1α	F:TACACCACGCAAAAGAGGCT<br>R:CTTGCAAAGCCACCTCAACC	199
GAPDH	F:TCTTGTCCGTGTGTGACGAG<br>R:TCTGCGAGTATCCCCACTCA	186
CYP	F:GATGGAAGAGGCGGGGAATC<br>R:TTTGGTGTTCCACTCTGCGT	199
TUA	F:TCTCACGCATCGACCACAAA<br>R:TCTTCCTCGTCGTCAACACC	195
TUB	F:CCAGGGGTCTGAAAATGGCT<br>R:TTTCAGCCTCGGTGAACTCC	194
FAD2	F:GTGAACGGGTTCCTCGTCTT<br>R:GGTAGTGCGGCATGGTAGAG	199
FAD3	F;CAAGCTCTACGGCGTACCAT<br>R:GGGTGCCAATATCGTGGTGA	191
DGAT	F:AAGCTCGTTTCCTACGCACA<br>R:CAACCCTTCCGAATGCAAGC	200
PDAT	F:GAGGCGATAACCGGACCAAT<br>R:GCCTCTTGTAAACTTCGCCG	196
LPCAT1.1	F:AGGATCTGCGTGAAGCACAA<br>R:AGGGAGAGGGAGACAATCCC	189
LPCAT1.2	F:ATGGAGCTGCCGGAGATG<br>R:CGAGAGGAAAGCTCCTGTAGCG	183
